# A highly specific and sensitive massive parallel sequencer-based test for somatic mutations in non-small cell lung cancer

**DOI:** 10.1371/journal.pone.0176525

**Published:** 2017-04-27

**Authors:** Yoshiaki Inoue, Jun Shiihara, Hitoshi Miyazawa, Hiromitsu Ohta, Megumi Higo, Yoshiaki Nagai, Kunihiko Kobayashi, Yasuo Saijo, Masanori Tsuchida, Mitsuo Nakayama, Koichi Hagiwara

**Affiliations:** 1 Graduate School, Saitama Medical University, Moroyama, Saitama, Japan; 2 Department of General Thoracic Surgery, Saitama Medical Center, Kawagoe, Saitama, Japan; 3 Department of Respiratory Medicine, Graduate School of Medicine, Tohoku University, Sendai, Miyagi, Japan; 4 Department of Respiratory Medicine, Saitama Medical University, Moroyama, Saitama, Japan; 5 Department of Respiratory Medicine, Jichi Medical University, Saitama, Saitama, Japan; 6 Clinical Laboratories for Cardiovascular Diseases, Jichi Medical University, Saitama, Saitama, Japan; 7 Department of Respiratory Medicine, Saitama International Medical Center, Hidaka, Saitama, Japan; 8 Department of Medical Oncology, Graduate School of Medical and Dental Sciences, Niigata University, Niigata, Niigata, Japan; 9 Department of Thoracic and Cardiovascular Surgery, Graduate School of Medical and Dental Sceinces, Niigata University, Niigata, Niigata, Japan; 10 Division of Pulmonary Medicine, Department of Medicine, Jichi Medical University, Shimotsuke, Tochigi, Japan; Advanced Centre for Treatment Research and Education in Cancer, INDIA

## Abstract

Molecular targeting therapy for non-small cell lung cancer (NSCLC) has clarified the importance of mutation testing when selecting treatment regimens. As a result, multiple-gene mutation tests are urgently needed. We developed a next-generation sequencer (NGS)-based, multi-gene test named the MINtS for investigating driver mutations in both cytological specimens and snap-frozen tissue samples. The MINtS was used to investigate the *EGFR*, *KRAS*, *BRAF* genes from DNA, and the *ERBB2*, and the *ALK*, *ROS1*, and *RET* fusion genes from RNA. We focused on high specificity and sensitivity (≥0.99) and even included samples with a cancer cell content of 1%. The MINtS enables testing of more than 100 samples in a single run, making it possible to process a large number of samples submitted to a central laboratory, and reducing the cost for a single sample. We investigated 96 cytological samples and 190 surgically resected tissues, both of which are isolated in daily clinical practice. With the cytological samples, we compared the results for the *EGFR* mutation between the MINtS and the PNA-LNA PCR clamp test, and their results were 99% consistent. In the snap-frozen tissue samples, 188/190 (99%) samples were successfully analyzed for all genes investigated using both DNA and RNA. Then, we used 200 cytological samples that were serially isolated in clinical practice to assess RNA quality. Using our procedure, 196 samples (98%) provided high-quality RNA suitable for analysis with the MINtS. We concluded that the MINtS test system is feasible for analyzing “druggable” genes using cytological samples and snap-frozen tissue samples. The MINtS will fill a needs for patients for whom only cytological specimens are available for genetic testing.

## Introduction

Recent progress in molecular targeting therapy for non-small cell lung cancer (NSCLC) has clarified the importance of mutation testing when selecting treatment regimens [1–3]. Accordingly, tests for epidermal growth factor receptor (*EGFR*) mutations [4, 5] and the fusion gene of the anaplastic lymphoma receptor kinase (*ALK*) [6, 7] have been introduced in clinical practice in many countries [8–10]. Drugs targeting several other cancer driver genes [11] have entered the market, and many others will follow in the near future. Thus, there is an urgent need for the development of multiple-gene mutation tests that can simultaneously screen all mutations that are relevant to the drugs currently available (i.e., “druggable” mutations). The massive parallel sequencer is an attractive instrument for the development of such tests.

The types of NSCLC specimens submitted to genetic testing differ among counties. Formalin-fixed, paraffin-embedded (FFPE) tissue specimens are utilized in many counties, whereas cytological specimens account for a significant portion of samples in others. DNA from plasma is increasingly used as an alternative source of tumor DNA; however, its ultimate utility still requires investigation [12,13]. Japan is one of the counties where cytological samples isolated during endoscopic examination represent a significant portion of samples, accounting for 1/3 of the NSCLC samples submitted to genetic testing [9]. The disadvantage of using cytological samples include that the specimens contain fewer the cancer cells than FFPE samples and the histological type is not always evident. On the other hand, their advantages include that the presence of cancer cells is quickly confirmed and the samples can be readily submitted to genetic testing.

We hypothesized that a test that could utilize a small amount of DNA isolated from cytological samples would fill the unmet needs of patients from whom an insufficient amount of tumor tissue is available for mutation testing. In the current study, we developed a next-generation sequencer (NGS)-based, multi-gene test that can be used to investigate driver mutations in cytological specimens. We set the following goals for the test system: (1) it can detect mutations in samples with a cancer-cell content of 1%. This level is based on our data that most cancer-positive cytological samples have a cancer cell content of 1% [14]. (2) It has a specificity and sensitivity >0.99. We considered that, from a clinical standpoint of view, 1 error out of 100 clinical samples is the allowable limit for the selection of treatment regimens. (3) It enables the testing of more than 100 samples in a single run, allowing the testing of a large number of samples and reducing the cost. We named the system is the MINtS (the Mutation Investigator using Next-era Sequencer) and investigated its performance using the “druggable” genes for which molecular targeting drugs are currently available or will be available in near future in Japan.

## Methods

### Samples

Cytological samples (bronchial washing or bronchial brushing isolated during bronchoscopy, debris from needle-aspirated biopsy, or pleural effusion) were divided into two portions. One was submitted to pathological examination to confirm the presence of cancer cells. The other was centrifuged within 30 min of isolation and stored in RNAlater stabilizing solution (ThermoFisher Scientific, Waltham, MA, USA). Surgically resected tissues were snap-frozen and stored at -80°C. Procedures for sample isolation and confirmation of cancer cells in the samples are shown in **S1 Fig** [9, 14]. DNA and RNA were isolated using a Maxwell RSC Instrument (Promega, Madison, WI, USA). Human genomic DNA isolated from immortalized B lymphocyte cell lines established from healthy Japanese volunteers was purchased from the Japanese Collection of Research Bioresources (Osaka, Japan). The PC-9 cell line that has an exon 19 deletion in the *EGFR* gene [E746–A750del (2235–2249delGGAATTAAGAGAAGC)] was purchased from the RIKEN BioResource Center (Ibaraki, Japan).

### Target driver genes and mutations

The MINtS can be used to evaluate the “druggable” driver genes that are relevant to the drugs currently available or will be available in the near future in Japan (**Table 1**).

**Table 1:** Mutations investigated.

DNA part of the MINtS			
	Gene	Involved exon	Mutation
	***EGFR***		
		**Exon 18**	G719S, G719C, G719A
		**Exon 19**	Exon 19 deletions*
		**Exon 20**	T790M, S768I
		**Exon 21**	L858R, L861Q
	***KRAS***		
		**Exon 2**	G12S, G12R, G12C, G12D, G12A, G12V, G13S, G13R, G13C, G13D, G13A, G13V
		**Exon 3**	G61K, Q61E, Q61R, Q61P, G61L, G61H
	***BRAF***		
		**Exon 11**	G466V, G469A, G469E, G469V
		**Exon 15**	D594G, D594V, G596R, V600E
	***ERBB2***		
		**Exon 20**	YVMA776-779ins, G776V-Cins, G776L-Cins, GSP781-783ins
RNA part of the MINtS			
	Gene	Fusion partners	
	***ALK***	*EML4*, *KIF5B*, *TFG*, *KLC1*	
	***RET***	*KIF5B*, *CCDC6*	
	***ROS1***	*TPM3*, *SDC4*, *CD74*, *EZR*, *LRIG3*, *SLC34A2*, *GOPC*	
	***OAZ1***^#^		

* A total of 55 different types of deletions were investigated.

# A housekeeping gene used as an internal control investigating the quality of RNA.

### PCR amplification and sequencing

**Fig 1:**
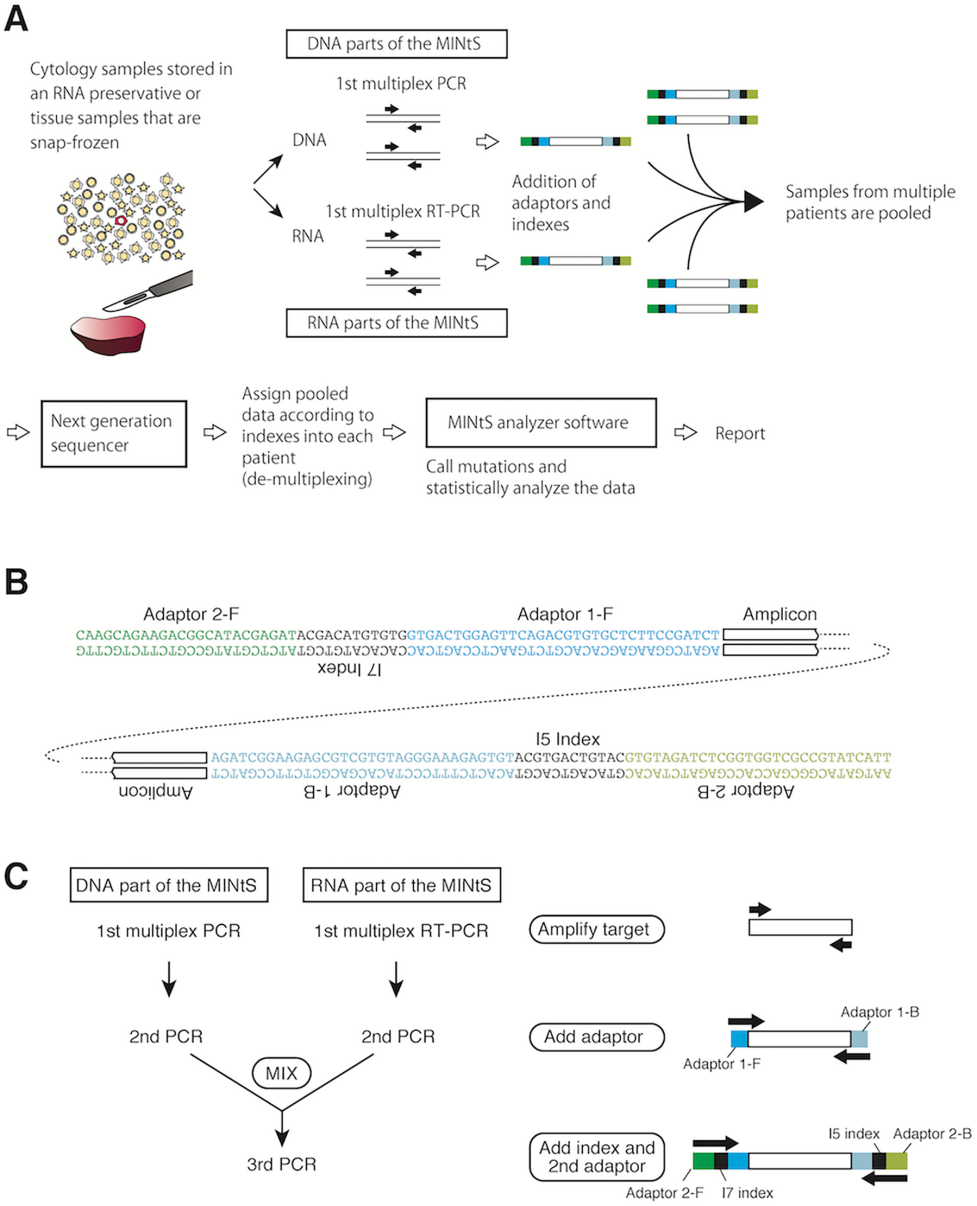
Strategy for the MINtS. (A) The procedures used for the MINtS. Samples isolated from a patient are immediately stored in an RNA preservative agent (cytological samples) or snap-frozen (tissue samples). DNA and RNA are prepared. The targets are amplified by multiplex PCR (DNA part of the MINtS) or by multiplex RT-PCR (RNA part of the MINtS). Adaptors required for loading on the next-generation sequencer (NGS) and indexes for discriminating individual samples are then appended by serial PCR reactions. Pooled samples are run on an NGS. The sequencing reads are de-multiplexed according to the indexes and assigned to each sample. The MINtS analyzer software performs the statistical analysis and identifies samples containing mutant genes. (B) Structure of the amplicons. Adaptor sequences are required for loading on the sequencer, whereas the indexes are used for associating each amplified DNA molecule to each sample. (C) Three serial PCR reactions for constructing amplicons. The 1st and the 2nd PCRs are separately performed for the DNA and RNA parts of the MINtS. The 3rd PCR is performed after mixing the two 2nd PCR reactions. The 48 different indexes on each side are able to discriminate 48^2^ = 2304 samples.

DNA was subjected to multiplex PCR to amplify the mutation hotspots. Thereafter, adaptors and indexes were added by two additional rounds of PCR (**Fig 1**). Here, adaptors are short stretches of nucleotides utilized by the MiSeq platform (Illumina Inc., San Diego, CA, USA) for the sequencing reaction. Indexes are 12-bp-long stretches of nucleotides we designed. Different indexes were attached to the amplicons from different patients, and thus, each amplified fragment could be traced back to the patient from whom it was derived (see **S1 Table** for PCR primers, **S2 Table** for RT-PCR primers, **S3 Table** for the index sequences). The final PCR products from multiple patients were mixed and sequenced on the MiSeq platform (**Fig 1**).

The 1st multiplex PCR and the 2nd PCR for the DNA part of the MINtS: First, 10 ng of genomic DNA was amplified in a 25-μL solution containing the primers for the DNA part of the MINtS (9 primers pairs, 250 nM each; **S1 Table**), 1× buffer for KOD Plus version 2, 200 nM dNTPs, 1 mM MgSO_4_, and 0.5 units of KOD Plus DNA polymerase (Toyobo; Osaka, Japan). The PCR cycling program was a 94°C hold for 120 s followed by 34 cycles of 94°C for 15 s, 62°C for 30 s, and 68°C for 30 s. For the 2nd PCR, 1 μL of the 1st multiplex PCR reaction was added to a 24-μL solution containing the 2nd PCR primers for the DNA part of the MINtS (25 nM each; **S1 Table**), 1× buffer for KOD Plus version 2, 200 nM dNTPs, 1 mM MgSO_4_, and 0.5 units of KOD Plus DNA polymerase. The PCR cycling program was a 94°C hold for 120 s followed by six cycles of 94°C for 15 s, 62°C for 30 s, and 68°C for 30 s.

The 1st multiplex RT-PCR and 2nd PCR for the RNA part of the MINtS: First, 10 ng of total RNA was reverse-transcribed from the oligo(dT) primers using the SuperScript VILO cDNA synthesis kit (ThermoFisher Scientific, Waltham, MA, USA) in a 20-μL reaction following the manufacturer’s instructions. Then, 1 μL of the reaction was transferred to a solution containing the primers for the RNA part of the MINtS (35 primers, 220 nM each; **S2 Table**), 1× buffer for KOD Plus version 2, 200 nM dNTPs, 1 mM MgSO_4_, and 0.5 units of KOD Plus DNA polymerase. The PCR cycling program was a 94°C hold for 120 s followed by 34 cycles of 94°C for 15 s, 62°C for 30 s, and 68°C for 30 s. For the 2nd PCR, 1 μL of the multiplex RT-PCR reaction was added to a 24-μL solution containing the 2nd PCR primers for the RNA part of the MINtS (25 nM each; **S2 Table**), 1× buffer for KOD Plus version 2, 200 nM dNTPs, 1 mM MgSO_4_, and 0.5 units of KOD Plus DNA polymerase. The PCR cycling program was a 94°C hold for 120 s followed by six cycles of 94°C for 15 s, 62°C for 30 s, and 68°C for 30 s.

In the 3rd PCR, the two 2nd PCR reactions (from the DNA part and the RNA part) derived from a single sample were mixed and amplified. The indexes were selected so that the combination of the indexes was unique for the sample, thereby allowing identification of each patient from whom a sequencing read originated. Here, 0.5 μL each of the two 2nd PCR reactions were added to a 24-μL solution containing the 3rd PCR primers (100 nM each; **S3 Table**), 1× buffer for KOD Plus version 2, 200 nM dNTPs, 1 mM MgSO_4_, and 0.5 units of KOD Plus DNA polymerase. The PCR cycling program was a 94°C hold for 120 s followed by six cycles of 94°C for 15 s, 62°C for 30 s, and 68°C for 30 s.

The three PCR products from multiple samples were mixed. Amplified DNA was purified using the Agencourt AMPure XP system (Beckman Coulter, Brea, CA, USA) and the final DNA concentration was adjusted to 4 ng/μL. Ten microliters of the DNA solution and 10 μL of sodium hydrate solution (0.2 M) were mixed, kept at room temperature for 5 min, and neutralized by adding 980 μL of hybridization buffer (HT1: Illumina), and 600 μL of this mixture was subjected to pair-end nucleotide sequencing using the MiSeq Reagent kit V3 (Illumina).

### Statistical analysis

**Fig 2:**
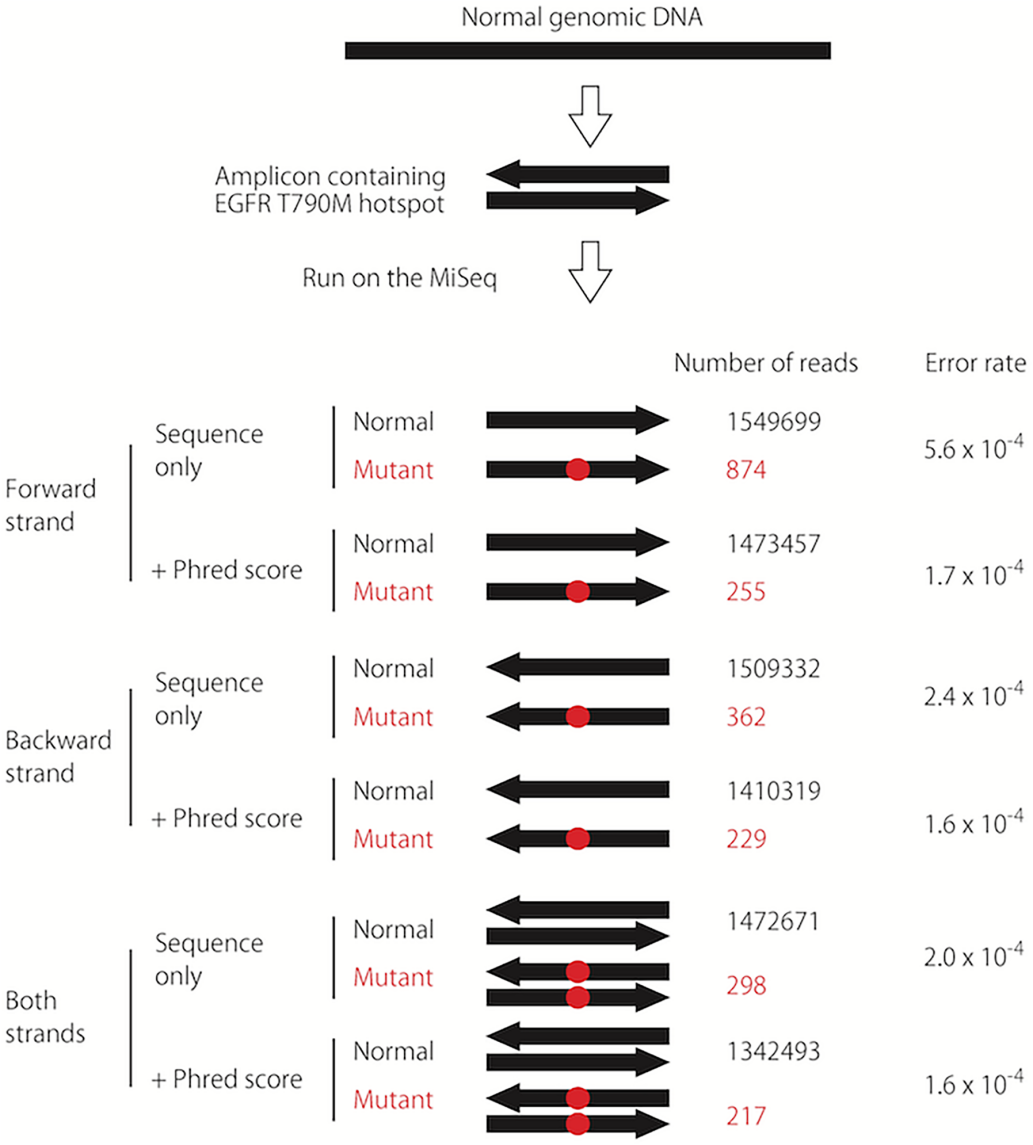
Determination of the false-positive rate. Normal genomic DNA isolated from immortalized lymphocytes (n = 40) was subjected to the MINtS, and the number of mutant reads was counted. The results for the *EGFR* T790M mutation are shown. By sequencing both strands and selecting for a Phred score >30 (i.e., calculated error rate of 10^−3^) [15] low false-positive rate sufficient for the highly specific detection of mutations was attained. The false-positive rates for the other hotpots are shown in **S4 Table.**

Rates for detection errors, the de-multiplexing errors, and the carry-over errors (see the Results section) were obtained (**S3 Table and S2 Fig**). MINtS analyzer software was used to select the reads with good quality (Phred score >30 and sequence data for both DNA strands matched). It then was used count the numbers of reads with normal or mutant sequences. Next, it was used to perform a statistical analysis based on the false-positive (**Fig 2 and S4 Table**) de-multiplexing error (**S3 Table**) rates. Finally, a diagnosis of either “negative for mutation” or “positive for mutation” was made if the statistical power was sufficient to identify the mutation in a sample with a cancer cell content of 1% (**S3 Fig**); otherwise, the result was undetermined. When the result was “positive for mutation,” the cancer cell content was calculated assuming that cancer cells are diploid.

### MINtS analyzer software

**Fig 3:**
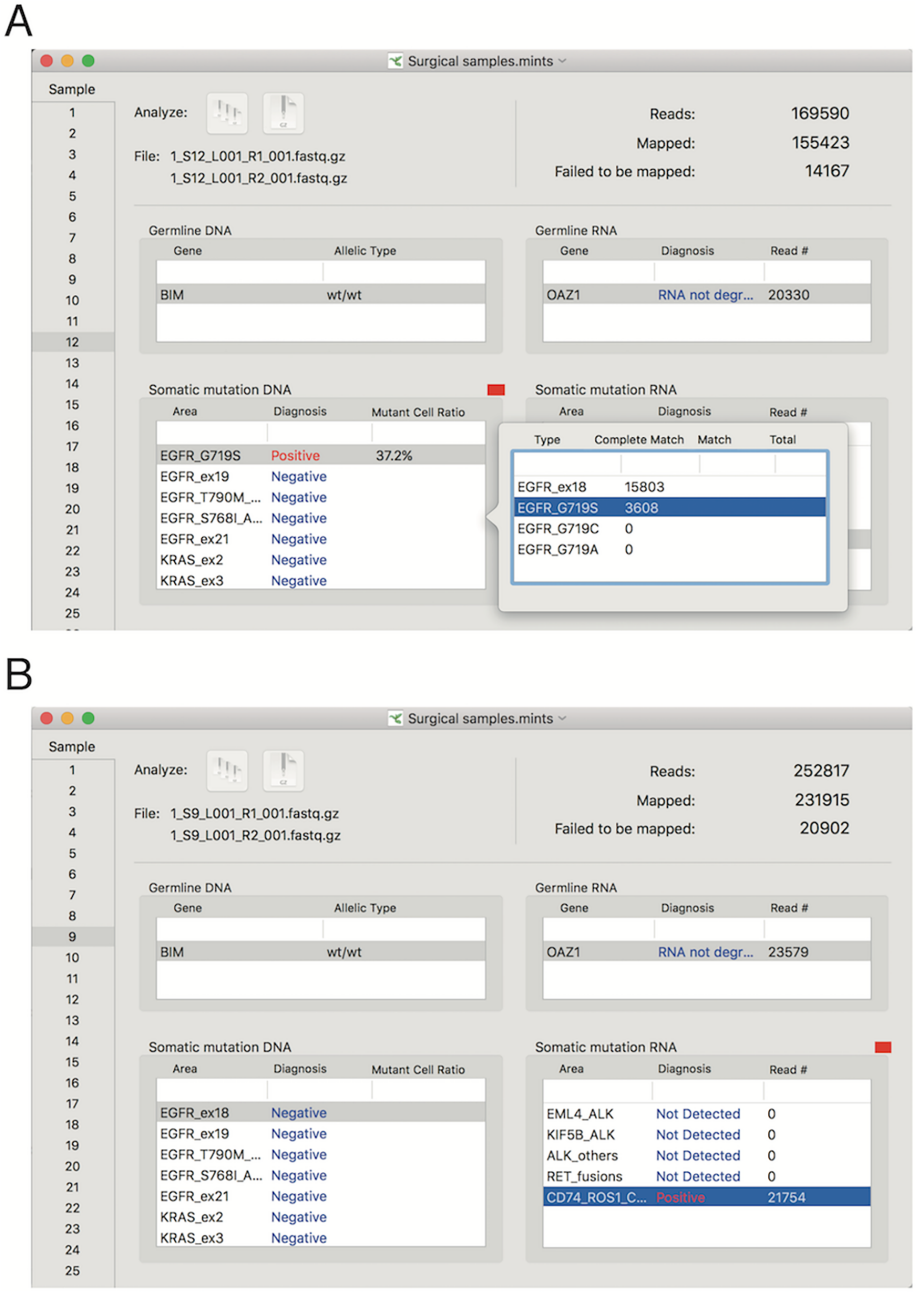
Screen shots of the MINtS analyzer software. Screen shots of samples with the G719S *EGFR* mutation (A) or *CD74*-*ROS1* fusion gene (B).

We upload the MINtS analyzer software and the sample fastq files to our website (http://www.hhanalysis.com) for download. The software was developed for the statistical analysis as stated above. It loads the fastq files that are output from the MiSeq, analyzes the data, and outputs the result using a graphical interface (**Fig 3**). The MINtS analyzer runs on the MacOS X and is available on request.

## Results

### Strategy overview

An amplicon-sequencing strategy was adapted for the MINtS (**Fig 1**). To increase the number of samples that can be simultaneously analyzed, all driver genes directly relevant to clinical practice were included [16,17]. Using DNA, the MINtS was used to investigates *EGFR*, the Kirsten rat sarcoma viral oncogene homolog gene (*KRAS*) [18], the v–raf murine sarcoma viral oncogene homolog B1 gene (*BRAF*) [19–21], and the erb-b2 receptor tyrosine kinase 2 gene (*ERBB2*) [22–24]; the target regions were amplified by multiplex PCR (the DNA part of the MINtS). Using RNA, the MINtS was used to investigate the *ALK*, ROS proto-oncogene 1, receptor tyrosine kinase (*ROS1*) [25, 26], and ret proto-oncogene (*RET*) fusion genes [25, 27] as well as the ornithine decarboxylase antizyme 1 (*OAZ1*) housekeeping gene; the targets were amplified by multiplex RT-PCR (the RNA part of the MINtS). The *OAZ1* gene was chosen as the RNA internal control [28] because it is evenly expressed at a low level in many cells [29]. Then the index sequences were added to both ends of each amplicon for discriminating the multiplicity of samples. The final PCR products were combined and run on the MiSeq next-generation sequencer (NGS). According to the indexes, the reads obtained were de-multiplexed and assigned to each sample. MINtS analyzer software was used to identify the reads for the *EGFR*, *KRAS*, *BRAF*, and *ERBB2* genes; the *ALK*, *ROS1*, and *RET* fusion genes; and the *OAZ1* housekeeping gene. The MINtS analyzer was then used to perform a statistical analysis and identify samples carrying a mutated gene.

### Reduction of errors

We found three major types of errors: (1) detection errors, (2) de-multiplexing errors, (3) and carry-over errors. Limiting these errors was vital for constructing a highly sensitive and specific multigene test.

Detection errors consist of DNA polymerase errors and MiSeq sequencer errors. DNA polymerase mistakenly incorporates incorrect nucleotides at mutation hotspots, thereby artificially producing a mutant sequence. The MiSeq sequencer can mistakenly call wrong sequences at mutation hotspots, resulting in the detection of a mutant sequence even when sequencing normal DNA. We used three procedures to reduce these errors: (1) employed a high-fidelity DNA polymerase, KOD (Toyobo, Osaka, Japan), (2) read both strands of the amplified DNA in both strand, and (3) selected only the sequencing reads with high quality (Phred score >30, i.e., rate of erroneous nucleotide call <10^−3^). These procedures reduced the false-positive rate to <0.0005 per read (**S3 Table**).

De-multiplexing errors occur when the MiSeq sequencer misreads the index portion of the amplicon and assigns the read to the wrong sample. This type of error causes serious inter-sample contamination of the data. We designed a set of 48 indexes so that any two indexes were different at ≥4 nucleotide positions (**S4 Table**). This enabled the clear discrimination of two different indexes (**S2 Fig**). We then appended indexes to both ends of a DNA fragment. This enabled us to discriminate 48^2^ = 2304 patients.

Carry-over errors occur when a small amount of DNA remaining in the sequencer contaminates the next sequencing run. We found that these errors occurred even after an extensive washing of the MiSeq sequencer using hypochlorite [30]. We decided not to use the same indexes in two consecutive runs because we had 2304 combinations of indexes, which was more than enough to perform this procedure. As a result, the carry-over errors disappeared.

### Statistical analysis

The successful limitation of the three types of errors (detection, de-multiplexing, and carry-over errors) enabled the detection of mutations with a specificity and sensitivity >0.99 or even >0.999 when a sufficient number of reads was obtained for a single hot spot. The algorithm used to identify the presence of mutations and the number of reads required for specific decisions are summarized in **S3 Fig**. The algorithm was implemented in the MINtS analyzer software and was used in all subsequent analyses.

### Performance using DNA isolated from cytological samples

We investigated 96 cytological samples randomly selected from our archives. They were isolated in daily clinical practice and had been tested for the *EGFR* mutation using the PNA-LNA PCR clamp method, which has been widely used in clinical practice in Japan and can detect mutated *EGFR* in a sample with a cancer cell content of 1% [9, 14, 28, 30, 31, 32]. Therefore, the results for the *EGFR* mutation can be compared between the PNA-LNA PCR clamp method and the MINtS. All samples were tested using the MINtS in a single sequencing run. Definite results were obtained for 95 samples; one sample was not amplified by PCR (**S4 Table**). The results from the two methods matched except for one, which had the lowest calculated cancer cell content of 0.8%. We concluded that the discrepancy was due to a false-negative result in the PNA-LNA PCR clamp method.

### Performance using DNA and RNA isolated from snap-frozen, surgically resected tissues

We then investigated 190 NSCLC samples that were surgically resected and snap-frozen. These tissues were expected to provide good quality DNA and RNA, and both were suitable for investigating the performance of the MINtS. The samples were sequenced in two separate runs (approximately 95 samples per each run). The results (**Table 2**) show that mutations were detected according to the frequencies as reported for NSCLC [11]. In two samples, the DNA or RNA was degraded. Even when many samples were run together, a sufficient number of reads was obtained for each sample (**S6 Table**). This enabled detection with a sensitivity and specificity >0.99.

**Table 2:** Results for the surgically resected samples.

			Number	Percentage*
DNA part of MINtS				
	*EGFR* mutations			
		Sensitive mutations	39	20.7
		Minor mutations	5	2.7
		With T790M	2	1.1
	*KRAS* mutations			
		Codons 12 and 13	24	12.8
		Codon 61	2	1.1
	*BRAF* mutations		2	1.1
	*ERBB2* mutations		3	1.6
RNA parts of MINtS				
	*ALK* fusion genes		3	1.6
	*ROS1* fusion genes		2	1.1
	*RET* fusion genes		2	1.1
Mutation not detected			104	55
DNA or RNA degraded			2	

A total of 190 surgically resected NSCLC samples were snap-frozen. DNA and RNA were obtained from all but two samples and amplified by PCR or RT-PCR to give a sufficient number of reads to attain a specificity and sensitivity >0.999 for the DNA part of the MINtS. Detailed data are presented in the **S5 Table**.

* The percentage was calculated for the samples in which DNA and RNA was not degraded (n = 188).

### Integrity of the RNA in the cytological samples

One of the most important prerequisites for performing the MINtS is the integrity of the RNA in the clinical samples. In the RNA part of the MINtS, specificity was considered to be very high because it is rare to produce the sequences of the fusion gene with an artifact. Attaining high sensitivity was a challenge. The results from the snap-frozen tissues suggested that the number of fusion gene reads was >0.4-fold of that of the *OAZ1* housekeeping gene. By assuming this value, 1150 reads for the *OAZ1* gene were calculated to enable the detection of the fusion gene with a sensitivity of 0.99 from samples with a cancer cell content of 1%. We investigated 200 serial cytological samples according to **S1 Fig** using two separate MiSeq runs. Ninety-eight percent of the samples (n = 196) provided enough reads for the *OAZ1* gene, attaining a sensitivity of 0.99 (**Table 3**). We concluded that cytological samples were suitable for testing RNA-fusion genes using the MINtS.

**Table 3:** Number of reads for the *OAZ1* housekeeping gene in 200 serial samples.

Sample with bad RNA (<1150 *OAZ1* reads)	Sample with good RNA (1151≤ *OAZ1* reads)
4	196
Details	
<1150 reads	4
1151–5000 reads	12
5000–10000 reads	28
10000–20000 reads	51
20000≤ reads	105

Based on the result of the present study, we are now conducting a clinical trial (clinical trial number UMIN000015665) in which 3000 cytological samples will be collected to evaluate the performance of the MINtS.

## Discussion

In the current study, we developed a highly sensitive and specific mutation test that can simultaneously investigate multiple “druggable” somatic mutations using the MiSeq massive parallel sequencer.

When a small number of mutant reads and a large number of normal reads are obtained for a sample, it is difficult to determine whether the mutant reads derive from cancer cells present in a small percentage or result from an artifact such as a de-multiplexing error. This was the major challenge to overcome in the DNA part of the MINtS. The use of a high-fidelity DNA polymerase, KOD, and a strategy involving sequencing both strands enabled us to distinguish between these two situations (**Fig 2**). Then, we increased the number of samples, but a new problem emerged. We initially used index sequences (eight nucleotides for both sides of the amplicon) from the Illumina’s TruSeq DNA (HT) High Throughput kit, which was designed to simultaneously run 96 samples. However, the inclusion of even a single sample with a mutated *EGFR* resulted in a significant number of the mutant reads in multiple samples due to de-multiplexing errors. Moreover, the inclusion of even a single sample with the mutated *EGFR* in a previous sequencing run resulted in a significant number of the same mutant reads in the next sequencing run, probably due to a small amount of DNA that remained in the circuit of the MiSeq. The extensive washing of the circuit with hypochlorous acid between two runs could not remove the contamination. We decided to use longer index sequences (12 nucleotides for both sides of the amplicon, shown in **S3 Table**), which solved the problem.

The recovery of high-quality RNA from clinical samples was considered the major challenge in the RNA part of the MINtS. However, we found that using the procedure described in **S1 Fig** consistently provided good quality RNA suitable for amplifying the *OAZ1* housekeeping gene, which is expressed in a relatively low amount. The amplicon size of many of the fusion genes in the MINtS was designed to be similar to that of *OAZ1*. We registered 43 different types for the fusion genes. We have not yet encountered all of these types in clinical samples, but we predict that the MINtS will be able to detect many of them. However, this prediction is pending confirmation by a clinical trial.

In a multi-gene test, the false-positives that occur for each mutation hotspot accumulate and comprise the false-positives for the entire test. Assuming a false-positive rate for each hotpot of 0.01, in a multi-gene test investigating 10 hotspots, the false-positive rate of the entire test becomes 0.1. Thus the MINtS must reduce the false-positive rate for each hotspot well below 0.01 to attain a specificity of 0.99 for the entire test. The low rate of false-positive reads (**Fig 2 and S4 Table**) enabled us to attain a specificity of 0.999 for each hotspot by obtaining 6241 reads (**S3 Fig**). Because the MiSeq Reagent kit V3 provides 10^7^ to 10^8^ reads for each run, this requirement was not difficult to meet.

In multi-gene tests investigating only driver genes, such as the MINtS, the false-negative rate does not accumulate because the driver gene is mutually exclusive, and thus, at most only a single hotspot is positive for a mutation, irrespective of the number of hotspots being tested. Therefore, the reduction of the false-negative rate was not a difficult problem to overcome for the MINtS.

The MINtS system has the capacity to investigate additional mutation hotspots. Our preliminary data suggested that 15 amplicons could be safely amplified in a single tube (data not shown). Alternatively, adding another multiplex PCR reaction or multiplex RT-PCR reaction (**Fig 1**) will enable the addition of multiple hotspots to the test. Several years ago, we considered that the number of “druggable” mutations increases rapidly. However, it stays within a range that the MINtS can manage, at least for the treatment of NSCLC. We conclude that the MINtS is feasible for clinical practice applications.

In the current study, we established a mutation test, the MINtS, for investigating multiple somatic mutations with a specificity and sensitivity >0.99. This was achieved by reducing the three types of common errors. The MINtS will provide a framework for mutation tests utilizing cytological samples and will fill the unmet needs of the patients from whom the isolation of tissue samples is difficult.

## Supporting information

S1 Fig Preparation of samples with a confirmed presence of cancer cells. (TIFF)

S2 Fig Measurement of the de-multiplexing error rate. Immortalized lymphocyte DNA (wild type *EGFR*; 20 samples) and PC-9 DNA [heterozygous for the *EGFR* exon 19 deletion E746–A750del (2235–2249delGGAATTAAGAGAAGC); 20 samples] were run on the MINtS. PC-9 DNA has a single copy of the mutated *EGFR* gene. The reads that were assigned to the normal lymphocyte DNA but have the mutated *EGFR* sequence (shown in red) were due to the de-multiplexing errors, for which the rate was 3.8 ×10^−5^.
(TIFF)

S3 Fig Statistical analysis. We prioritized specificity over sensitivity. (A) *f*(*n*,*p*) is the distribution of the number of falsely mutant reads for a normal sample, where *n* is the number of reads, and *p* is the rate of false-positive reads. (B) *g*(*n*,0.005) is the distribution of the number of mutant read for a sample with a 1% cancer cell content (the frequency of the mutant allele is 0.005). (C) The area in black is the false-positive rate, whereas that in gray is the false-negative rate. The number of reads (*n*) must be sufficient to attain “the threshold determining sensitivity ≤ the threshold determining specificity”. (D) Criteria for deciding the presence of a mutation. (E) The number of reads (*n*) required for attaining a sensitivity and specificity ≥0.99 or ≥0.999 according to the rate of false-positive reads.
(TIFF)

S1 Table Multiplex RT-PCR primer sequences. Primers for the 1st and the 2nd PCR are shown. Sequences for the adaptors 1-F and 1-B are colored in blue and light blue, respectively.
(DOC)

S2 Table Multiplex RT-PCR primer sequences. Primers for the 1st and the 2nd PCR are shown. Sequences for the adaptors 1-F and 1-B are colored in blue and light blue, respectively.
(DOCX)

S3 Table 3rd PCR primer sequences. Primers for the 3rd PCR are shown. Sequences for the adaptors 1-F and 1-B are colored in blue and light blue, respectively, whereas the sequences for the adaptors 2-F and 2-B are shown in green and yellow green, respectively. Index sequences are shown in black. For an individual sample, one 5ʹ primer and one 3ʹ primer were selected so that each sample had a different combination of indexes. Here, any two I5 indexes or any two I7 indexes differ in at least four positions. When entering the index sequence in the for the MiSeq SampleSheet.csv file, the anti-sense of the 5ʹ primer index sequence is entered as the I7 index, and the sense of the 3ʹ primer index sequence is entered as the I5 index. For example, when using 5ʹK301 and 3ʹH501, enter “CACACATGTCGT” as the I7 index and the “GTACAGTCACGT” as the I5 index.
(XLSX)

S4 Table False-positive rates. The false-positive rates for each hotspots when both strand were sequenced and only the reads with a Phred score >30 were selected.
(DOCX)

S5 Table Mutation testing using DNA from the cytological samples and comparison with the results obtained by the PNA-LNA PCR clamp method. The results were sorted first by the type of mutation and then the calculated cancer-cell content.
(DOCX)

S6 Table DNA and RNA testing using the surgically resected samples. The results are presented by the type of mutations, and sorted by either the percentage of the cells harboring the mutation in the samples or the number of reads for the *OAZ1* gene (DOC).
(DOCX)
