# Supplementary figures and images for: A highly specific and sensitive massive parallel sequencer-based test for somatic mutations in non-small cell lung cancer
Source: PLoS One. 2017 Apr 27;12(4):e0176525. doi: 10.1371/journal.pone.0176525 (PMC5407820; doi:10.1371/journal.pone.0176525)

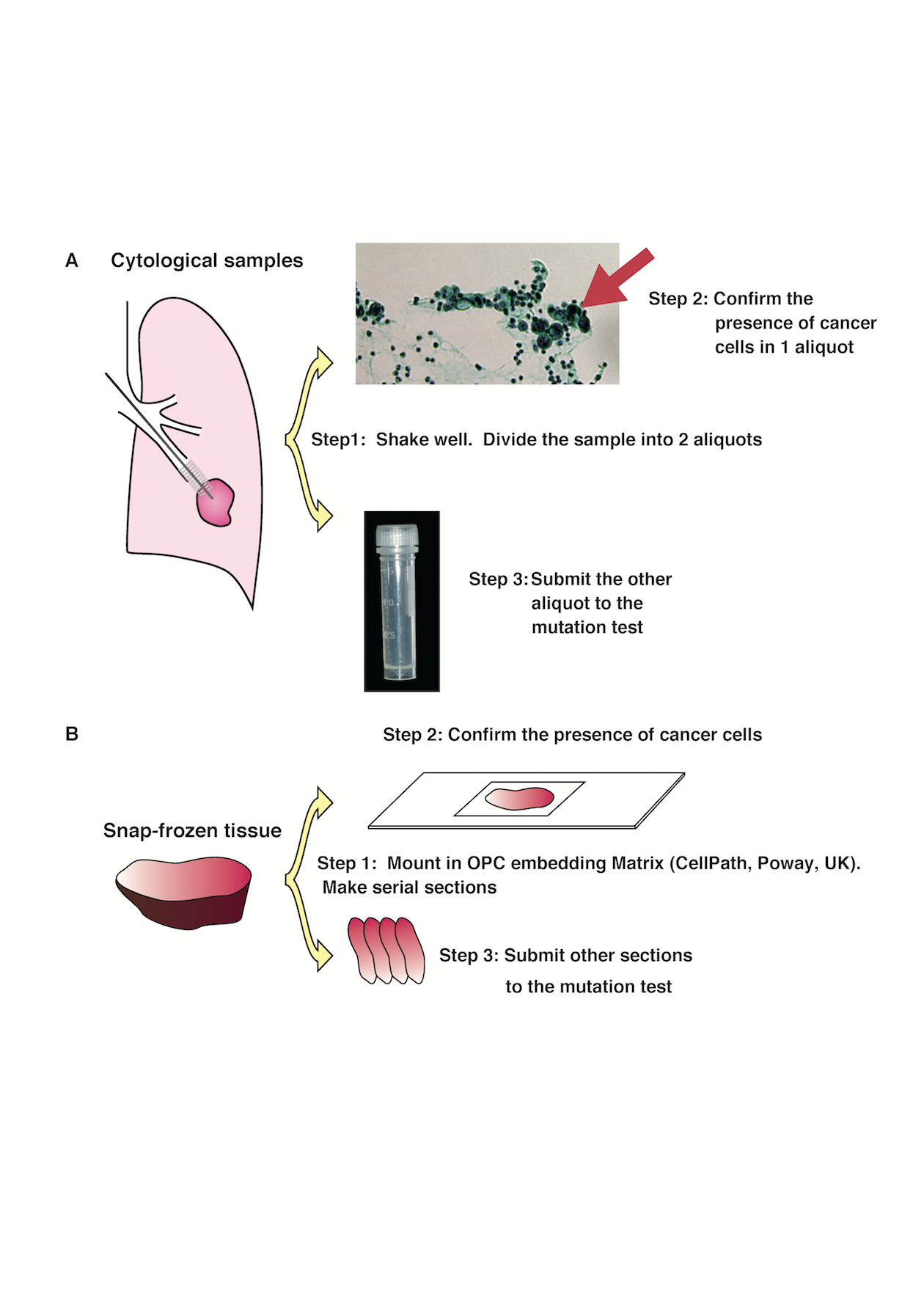

Supplement: S1 Fig — (TIFF) [file pone.0176525.s001.tiff]

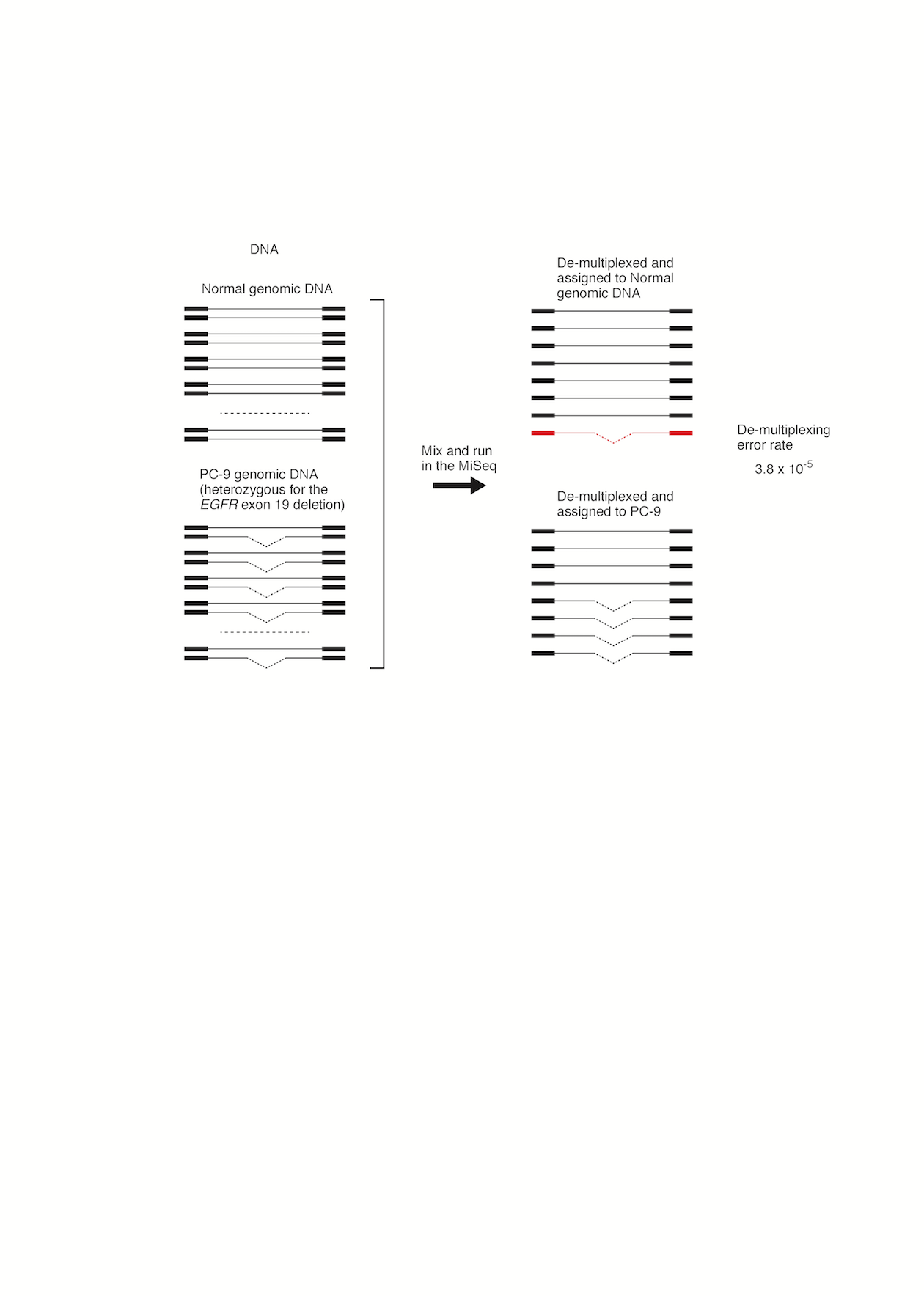

Supplement: S2 Fig — Immortalized lymphocyte DNA (wild type EGFR; 20 samples) and PC-9 DNA [heterozygous for the EGFR exon 19 deletion E746–A750del (2235–2249delGGAATTAAGAGAAGC); 20 samples] were run on the MINtS. PC-9 DNA has a single copy of the mutated EGFR gene. The reads that were assigned to the normal lymphocyte DNA but have the mutated EGFR sequence (shown in red) were due to the de-multiplexing errors, for which the rate was 3.8 ×10^−5^. (TIFF) [file pone.0176525.s002.tiff]

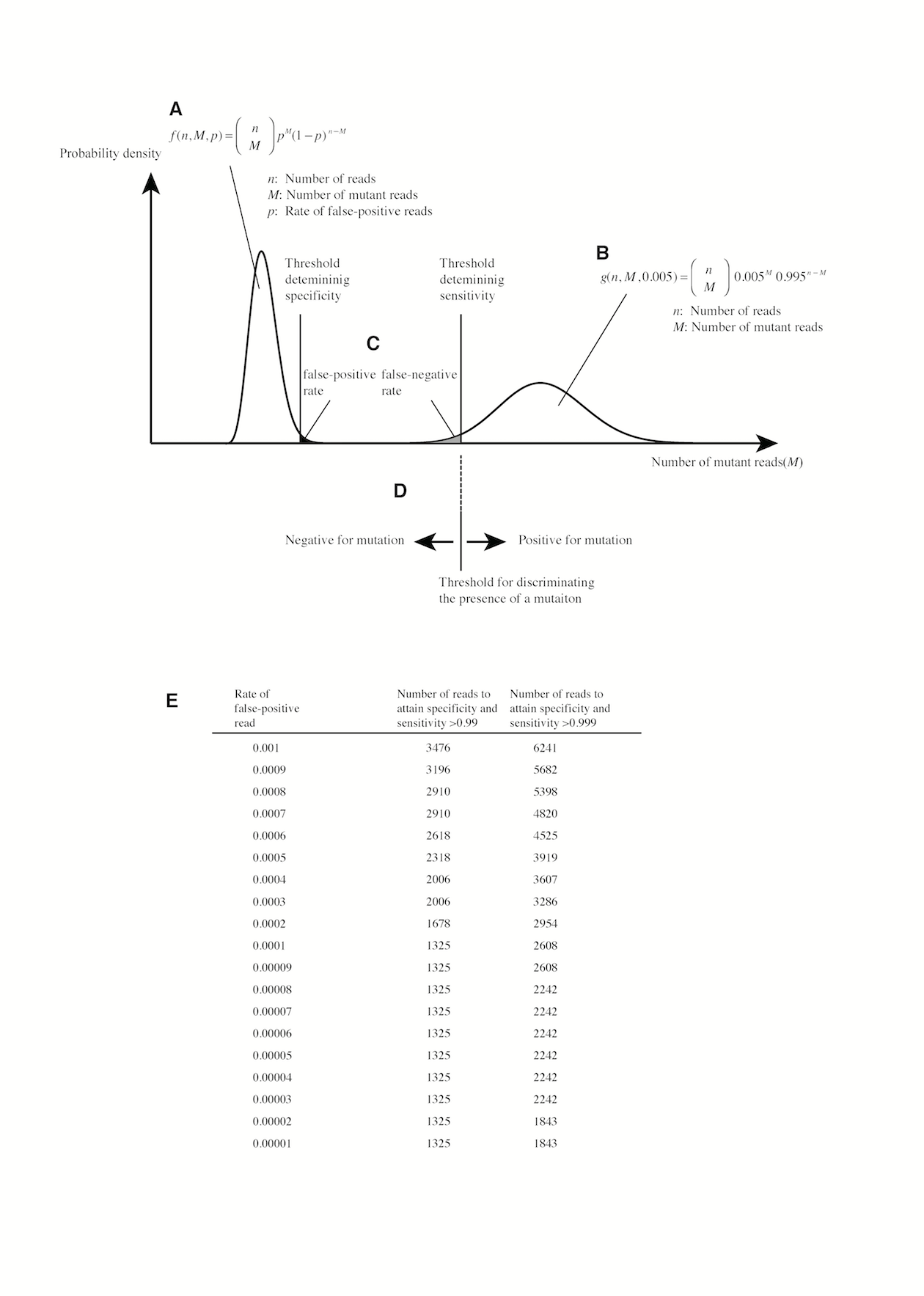

Supplement: S3 Fig — We prioritized specificity over sensitivity. (A) f(n,p) is the distribution of the number of falsely mutant reads for a normal sample, where n is the number of reads, and p is the rate of false-positive reads. (B) g(n,0.005) is the distribution of the number of mutant read for a sample with a 1% cancer cell content (the frequency of the mutant allele is 0.005). (C) The area in black is the false-positive rate, whereas that in gray is the false-negative rate. The number of reads (n) must be sufficient to attain “the threshold determining sensitivity ≤ the threshold determining specificity”. (D) Criteria for deciding the presence of a mutation. (E) The number of reads (n) required for attaining a sensitivity and specificity ≥0.99 or ≥0.999 according to the rate of false-positive reads. (TIFF) [file pone.0176525.s003.tiff]
