# Supplementary material for: A highly specific and sensitive massive parallel sequencer-based test for somatic mutations in non-small cell lung cancer
Source: PLoS One. 2017 Apr 27;12(4):e0176525. doi: 10.1371/journal.pone.0176525 (PMC5407820; doi:10.1371/journal.pone.0176525)
Supplement: S1 Table — Primers for the 1st and the 2nd PCR are shown. Sequences for the adaptors 1-F and 1-B are colored in blue and light blue, respectively. (DOC) [file pone.0176525.s004.doc]

| 1st multiplex PCR primers for the DNA part of the MINtS | | |  |  |
| --- | --- | --- | --- | --- |
|  |  |  |  |  |
|  | Gene | Name | Sequence | Final concentration (nmol/L) |
|  | EGFR |  |  |  |
|  |  | EGFR-ex18-1F | 5'-CTTACACCCAGTGGAGAAGCTC-3' | 160 |
|  |  | EGFR-ex18-1B | 5'-CCCACCAGACCATGAGAGG-3' | 160 |
|  |  | EGFR-ex19-1F | 5'-GCATGTGGCACCATCTCAC-3' | 120 |
|  |  | EGFR-ex19-1B | 5'-CTGAGGTTCAGAGCCATGGAC-3' | 120 |
|  |  | EGFR-ex20-1F | 5'-CATGCGTCTTCACCTGGAAGG-3' | 160 |
|  |  | EGFR-ex20-1B | 5'-CACACCAGTTGAGCAGGTACTG-3' | 160 |
|  |  | EGFR-ex21-1F | 5'-GCAGAGCTTCTTCCCATGATGATC-3' | 160 |
|  |  | EGFR-ex21-1B | 5'-CCTTACTTTGCCTCCTTCTGCA-3' | 160 |
|  | KRAS |  |  |  |
|  |  | KRAS-ex2-1F | 5'-AAGGCCTGCTGAAAATGACTG-3' | 120 |
|  |  | KRAS-ex2-1B | 5'-GAATGGTCCTGCACCAGTAATATG-3' | 120 |
|  |  | KRAS-ex3-1F | 5'-CAGGATTCCTACAGGAAGCAAGTAG-3' |  |
|  |  | KRAS-ex3-1B | 5'-GGCAAATACACAAAGAAAGCCCTC-3' | 240 |
|  | BRAF |  |  | 240 |
|  |  | BRAF-ex11-1F | 5'-GAAAACACTTGGTAGACGGGACTCG-3' | 120 |
|  |  | BRAF-ex11-1B | 5'-CACCACATTACATACTTACCATGCC-3' | 120 |
|  |  | BRAF-ex15-1F | 5'-GCTTGCTCTGATAGGAAAATGAGATCTACTG-3' | 120 |
|  |  | BRAF-ex15-1B | 5'-CCACAAAATGGATCCAGACAACTGTTC-3' | 120 |
|  | ERBB2 |  |  |  |
|  |  | ERBB2-ex20-1F | 5'-CCTCTCAGCGTACCCTTGTCC-3' | 160 |
|  |  | ERBB2-ex20-1B | 5'-CTCCGGAGAGACCTGCAAAGAG-3’ | 160 |
|  |  |  |  |  |
|  |  |  |  |  |
|  |  |  |  |  |
| 2nd PCR primers for the DNA part of the MINtS | | |  |  |
|  | Gene | Name | Sequence | Final concentration (nmol/L) |
|  | EGFR |  |  |  |
|  |  | EGFR-ex18-2F | 5'-CACGACGCTCTTCCGATCTCTTACACCCAGTGGAGAAGCTCC-3' | 25 |
|  |  | EGFR-ex18-2B | 5'-GACGTGTGCTCTTCCGATCTCCCACCAGACCATGAGAGGC-3' | 25 |
|  |  | EGFR-ex19-2F | 5'-CACGACGCTCTTCCGATCTGCATGTGGCACCATCTCACAATTG-3' | 25 |
|  |  | EGFR-ex19-2B | 5'-GACGTGTGCTCTTCCGATCTCTGAGGTTCAGAGCCATGGACC-3' | 25 |
|  |  | EGFR-ex20-2F | 5'-CACGACGCTCTTCCGATCTCATGCGTCTTCACCTGGAAGGG-3' | 25 |
|  |  | EGFR-ex20-2B | 5'-GACGTGTGCTCTTCCGATCTCACACCAGTTGAGCAGGTACTGG-3' | 25 |
|  |  | EGFR-ex21-2F | 5'-CACGACGCTCTTCCGATCTGCAGAGCTTCTTCCCATGATGATCT-3' | 25 |
|  |  | EGFR-ex21-2B | 5'-GACGTGTGCTCTTCCGATCTCCTTACTTTGCCTCCTTCTGCATGG-3' | 25 |
|  | KRAS |  |  |  |
|  |  | KRAS-ex2-2F | 5'-CACGACGCTCTTCCGATCTAAGGCCTGCTGAAAATGACTGAATATAAACTTG-3' | 25 |
|  |  | KRAS-ex2-2B | 5'-GACGTGTGCTCTTCCGATCTGAATGGTCCTGCACCAGTAATATGCAT-3' | 25 |
|  |  | KRAS-ex3-2F | 5'-CACGACGCTCTTCCGATCTCAGGATTCCTACAGGAAGCAAGTAGTAATTG-3' | 25 |
|  |  | KRAS-ex3-2B | 5'-GACGTGTGCTCTTCCGATCTGGCAAATACACAAAGAAAGCCCTCC-3' | 25 |
|  | BRAF |  |  |  |
|  |  | BRAF-ex11-2F | 5'-CACGACGCTCTTCCGATCTGAAAACACTTGGTAGACGGGACTCG-3' | 25 |
|  |  | BRAF-ex11-2B | 5'-GACGTGTGCTCTTCCGATCTCACCACATTACATACTTACCATGCCACTTT-3' | 25 |
|  |  | BRAF-ex15-2F | 5'-CACGACGCTCTTCCGATCTGCTTGCTCTGATAGGAAAATGAGATCTACTG-3' | 25 |
|  |  | BRAF-ex15-2B | 5'-GACGTGTGCTCTTCCGATCTCCACAAAATGGATCCAGACAACTGTTC-3' | 25 |
|  | ERBB2 |  |  |  |
|  |  | ERBB2-ex20-2F | 5'-CACGACGCTCTTCCGATCTCCTCTCAGCGTACCCTTGTCCC-3' | 25 |
|  |  | ERBB2-ex20-2B | 5'-GACGTGTGCTCTTCCGATCTCTCCGGAGAGACCTGCAAAGAGC-3' | 25 |
