# Supplementary material for: A highly specific and sensitive massive parallel sequencer-based test for somatic mutations in non-small cell lung cancer
Source: PLoS One. 2017 Apr 27;12(4):e0176525. doi: 10.1371/journal.pone.0176525 (PMC5407820; doi:10.1371/journal.pone.0176525)
Supplement: S2 Table — Primers for the 1st and the 2nd PCR are shown. Sequences for the adaptors 1-F and 1-B are colored in blue and light blue, respectively. (DOCX) [file pone.0176525.s005.docx]

| 1st multiplex RT-PCR primers for the RNA part of the MINtS | | |  |  |
| --- | --- | --- | --- | --- |
| 5' primer | |  |  |  |
|  | Gene | Name | Sequence | Final concentration (nmole/L) |
|  | EML4 |  |  |  |
|  |  | EML4-ex2-1F | 5'-GTCTTGCAATCTCTGAAGATCATG-3' | 220 |
|  |  | EML4-ex5-1F | 5'-TAATACCAAAAGTTACCAAAACTGCAGAC-3' | 220 |
|  |  | EML4-ex13-1F | 5'-GAGACTCAGGTGGAGTCATG-3' | 220 |
|  |  | EML4-ex14-1F | 5'-CTTTGTCAGATGAGAAATGGGATG-3' | 220 |
|  |  | EML4-ex17-1F | 5'-CACTGTGCAGATTTTCATCCAAG-3' | 220 |
|  |  | EML4-ex20-1F | 5'-CATCACACACCTTGACTGGTC-3' | 220 |
|  | KIF5B |  |  |  |
|  |  | KIF5B-ex2-1F | 5'-CAGTCAAGCACATCTCAAGAGC-3' | 220 |
|  |  | KIF5B-ex7-1F | 5'-GTAGTCCAGATGAAGTTATGGATACC-3' | 220 |
|  |  | KIF5B-ex11-1F | 5'-CTGCAGAACAGTGGAAAAAGAAG-3' | 220 |
|  |  | KIF5B-ex15-1F | 5'-CGAGCAGCTGAGATGATGG-3' | 220 |
|  |  | KIF5B-ex17-1F | 5'-GAAAAGACAGTTGGAGGAATCTG-3' | 220 |
|  |  | KIF5B ex22-1F | 5'-GCAAAAGAACTTCAGACTTTACAC-3' | 220 |
|  |  | KIF5B-ex24-1F | 5'-GCTATCAGCAAGAAGTAGATCGC-3' | 220 |
|  | TFG |  |  |  |
|  |  | TFG-ex4-1F | 5'-CAGGTGAAATATCTCCGTCGAG-3' | 220 |
|  | KLC1 |  |  |  |
|  |  | KLC1-ex9-1F | 5'-CAAGGAAAGTTCAAGCAAGCAG-3' | 220 |
|  | CCDC6 |  |  |  |
|  |  | CCDC6-ex1-1F | 5'-GACCTACAAACTGAAGTGCAAG-3' | 220 |
|  | TPM3 |  |  |  |
|  |  | TPM3-ex8-1F | 5'-GTAGCCAAGCTGGAAAAGAC-3' | 220 |
|  | SDS4 |  |  |  |
|  |  | SDC4-ex2-1F | 5'-CCTAGAAGGCCGATACTTCTC-3' | 220 |
|  |  | SDC4-ex4-1F | 5'-GTGTCCAACAAGGTGTCAATG-3' | 220 |
|  | SLC34A2 | |  |  |
|  |  | SLC34A2-ex4-1F | 5'-CTCTGTTTCTTCCAAGGGATTG-3' | 220 |
|  |  | SLC34A2-ex13_568-1F | 5'-CTGTCAAGGCTCCTGAGAC-3' | 220 |
|  | CD74 |  |  |  |
|  |  | CD74-ex6-1F | 5'-GCTCCTGTTTGAAATGAGCAG-3' | 220 |
|  | EZR |  |  |  |
|  |  | EZR-ex10-1F | 5'-AGAAGGAGGAGTTGATGCTG-3' | 220 |
|  | LRIG3 |  |  |  |
|  |  | LRIG3-ex16-1F | 5'-GAGACCAACTTGCCAGCAG-3' | 220 |
|  | GOPC |  |  |  |
|  |  | GOPC-1F | 5'-GTGGTAACCCTGGTGCTAGTTG-3' | 220 |
|  | OAZ1 (internal control) | | |  |
|  |  | OAZ1-1F | 5'-CAGTGAGAGTTCCAGGGTC-3' | 220 |
|  |  |  |  |  |
| 3' primer | |  |  |  |
|  | Gene | Name | Sequence | Final concentration (nmole/L) |
|  | ALK |  |  |  |
|  |  | ALK-ex20-1-1B | 5'-CATGGCTTGCAGCTCCTG-3’ | 220 |
|  |  | ALK-ex20-2-1B | 5'-GAGCTTGCTCAGCTTGTACTC-3' | 220 |
|  | RET |  |  |  |
|  |  | RET ex8-1B | 5'-CTTGCTGACTGCACAGGAC-3' | 220 |
|  |  | RET-ex11-1B | 5'-CGAGACGATGAAGGAGAAGAGG-3' | 220 |
|  |  | RET ex12-1B | 5'-CAAGTTCTTCCGAGGGAATTCC-3' | 220 |
|  | ROS1 |  |  |  |
|  |  | ROS1-ex32-1B | 5'-CTCCCTTCTAGTAATTTGGGAATGC-3' | 220 |
|  |  | ROS1-ex34-1B | 5'-GTAAGTATGAAACTTGTTTCTGGTATCC-3' | 220 |
|  |  | ROS1-ex35-1B | 5'-CACTGTCACCCCTTCCTTG-3' | 220 |
|  | OAZ1 (internal control) | | |  |
|  |  | OAZ1-1B | 5'-CGTTGGACGTTAGTTCCTCTG-3' | 220 |
|  |  |  |  |  |
|  |  |  |  |  |
|  |  |  |  |  |
| 2nd PCR primers for the RNA part of the MINtS | | |  |  |
| 5' primer | |  |  |  |
|  | Gene | Name | Sequence | Final concentration (nmole/L) |
|  | EML4 |  |  |  |
|  |  | EML4-ex2-2F | 5'-CACGACGCTCTTCCGATCTGTCTTGCAATCTCTGAAGATCATGTGGC-3' | 25 |
|  |  | EML4-ex5-2F | 5'-CACGACGCTCTTCCGATCTTAATACCAAAAGTTACCAAAACTGCAGACAAGC-3' | 25 |
|  |  | EML4-ex13-2F | 5'-CACGACGCTCTTCCGATCTGAGACTCAGGTGGAGTCATGCTTATATG-3' | 25 |
|  |  | EML4-ex14-2F | 5'-CACGACGCTCTTCCGATCTCTTTGTCAGATGAGAAATGGGATGTTATTAACTGG-3' | 25 |
|  |  | EML4-ex17-2F | 5'-CACGACGCTCTTCCGATCTCACTGTGCAGATTTTCATCCAAGTGG-3' | 25 |
|  |  | EML4-ex20-2F | 5'-CACGACGCTCTTCCGATCTCATCACACACCTTGACTGGTCC-3' | 25 |
|  | KIF5B |  |  |  |
|  |  | KIF5B-ex2-2F | 5'-CACGACGCTCTTCCGATCTCAGTCAAGCACATCTCAAGAGCAAG-3' | 25 |
|  |  | KIF5B-ex7-2F | 5'-CACGACGCTCTTCCGATCTGTAGTCCAGATGAAGTTATGGATACCATAG-3' | 25 |
|  |  | KIF5B-ex11-2F | 5'-CACGACGCTCTTCCGATCTCTGCAGAACAGTGGAAAAAGAAGTATGAAAAAG-3' | 25 |
|  |  | KIF5B-ex15-2F | 5'-CACGACGCTCTTCCGATCTCGAGCAGCTGAGATGATGGCATC-3' | 25 |
|  |  | KIF5B-ex17-2F | 5'-CACGACGCTCTTCCGATCTGAAAAGACAGTTGGAGGAATCTGTCGATG-3' | 25 |
|  |  | KIF5B-ex22-2F | 5'-CACGACGCTCTTCCGATCTGCAAAAGAACTTCAGACTTTACACAACCTGC-3' | 25 |
|  |  | KIF5B-ex24-2F | 5'-CACGACGCTCTTCCGATCTGCTATCAGCAAGAAGTAGATCGCATAAAG-3' | 25 |
|  | TFG |  |  |  |
|  |  | TFG-ex4-2F | 5'-CACGACGCTCTTCCGATCTCAGGTGAAATATCTCCGTCGAGAACTG-3' | 25 |
|  | KLC1 |  |  |  |
|  |  | KLC1-ex9-2F | 5'-CACGACGCTCTTCCGATCTCAAGGAAAGTTCAAGCAAGCAGAAACAC-3' | 25 |
|  | CCDC6 |  |  |  |
|  |  | CCDC6-ex1-2F | 5'-CACGACGCTCTTCCGATCTGACCTACAAACTGAAGTGCAAGGCAC-3' | 25 |
|  | TPM3 |  |  |  |
|  |  | TPM3-ex8-2F | 5'-CACGACGCTCTTCCGATCTGTAGCCAAGCTGGAAAAGACAATTGATG-3' | 25 |
|  | SDS4 |  |  |  |
|  |  | SDC4-ex2-2F | 5'-CACGACGCTCTTCCGATCTCCTAGAAGGCCGATACTTCTCCG-3' | 25 |
|  |  | SDC4-ex4-2F | 5'-CACGACGCTCTTCCGATCTGTGTCCAACAAGGTGTCAATGTCCAG-3' | 25 |
|  | SLC34A2 | |  |  |
|  |  | SLC34A2-ex4-2F | 5'-CACGACGCTCTTCCGATCTCTCTGTTTCTTCCAAGGGATTGGGAG-3' | 25 |
|  |  | SLC34A2-ex13_568-2F | 5'-CACGACGCTCTTCCGATCTCTGTCAAGGCTCCTGAGACCTTTG-3' | 25 |
|  | CD74 |  |  |  |
|  |  | CD74-ex6-2F | 5'-CACGACGCTCTTCCGATCTGCTCCTGTTTGAAATGAGCAGGCAC-3' | 25 |
|  | EZR |  |  |  |
|  |  | EZR-ex10-2F | 5'-CACGACGCTCTTCCGATCTAGAAGGAGGAGTTGATGCTGCG-3' | 25 |
|  | LRIG3 |  |  |  |
|  |  | LRIG3-ex16-2F | 5'-CACGACGCTCTTCCGATCTGAGACCAACTTGCCAGCAGATATTC-3' | 25 |
|  | GOPC |  |  |  |
|  |  | GOPC-2F | 5'-CACGACGCTCTTCCGATCTGTGGTAACCCTGGTGCTAGTTGCAAAG-3' | 25 |
|  | OAZ1 (internal control) | | |  |
|  |  | OAZ1-2F | 5'-CACGACGCTCTTCCGATCTCAGTGAGAGTTCCAGGGTCTCCC-3’ | 25 |
|  |  |  |  |  |
| 3' primer | |  |  |  |
|  | Gene | Name | Sequence | Final concentration (nmole/L) |
|  | ALK |  |  |  |
|  |  | ALK-ex20-1-2B | 5'-GACGTGTGCTCTTCCGATCTCATGGCTTGCAGCTCCTGG-3' | 25 |
|  |  | ALK-ex20-2-2B | 5'-GACGTGTGCTCTTCCGATCTGAGCTTGCTCAGCTTGTACTCAG　-3' | 25 |
|  | RET |  |  |  |
|  |  | RET-ex8-2B | 5'-GACGTGTGCTCTTCCGATCTCTTGCTGACTGCACAGGACAGG-3' | 25 |
|  |  | RET-ex11-2B | 5'-GACGTGTGCTCTTCCGATCTCGAGACGATGAAGGAGAAGAGG-3' | 25 |
|  |  | RET-ex12-2B | 5'-GACGTGTGCTCTTCCGATCTCAAGTTCTTCCGAGGGAATTCCC-3' | 25 |
|  | ROS1 |  |  |  |
|  |  | ROS1-ex32-2B | 5'-GACGTGTGCTCTTCCGATCTCTCCCTTCTAGTAATTTGGGAATGCC-3' | 25 |
|  |  | ROS1-ex34-2B | 5'-GACGTGTGCTCTTCCGATCTGTAAGTATGAAACTTGTTTCTGGTATCCAAAAATCATC-3' | 25 |
|  |  | ROS1-ex35-2B | 5'-GACGTGTGCTCTTCCGATCTCACTGTCACCCCTTCCTTGG-3' | |
|  | OAZ1 (internal control) | | |  |
|  |  | OAZ1-2B | 5'-GACGTGTGCTCTTCCGATCTCGTTGGACGTTAGTTCCTCTGTTACATTC-3' | 25 |
