# Supplementary material for: A highly specific and sensitive massive parallel sequencer-based test for somatic mutations in non-small cell lung cancer
Source: PLoS One. 2017 Apr 27;12(4):e0176525. doi: 10.1371/journal.pone.0176525 (PMC5407820; doi:10.1371/journal.pone.0176525)
Supplement: S4 Table — The false-positive rates for each hotspots when both strand were sequenced and only the reads with a Phred score >30 were selected. (DOCX) [file pone.0176525.s007.docx]

| 5' primer (for the I7 index) | | |  |
| --- | --- | --- | --- |
|  | Name | Sequence | Final concentration (nmol/L) |
|  | 5'K301 | CAAGCAGAAGACGGCATACGAGATACGACATGTGTGGTGACTGGAGTTCAGACGTGTGCTCTTCCGATCT | 100 |
|  | 5'K302 | CAAGCAGAAGACGGCATACGAGATGTAGTGCACACAGTGACTGGAGTTCAGACGTGTGCTCTTCCGATCT | 100 |
|  | 5'K303 | CAAGCAGAAGACGGCATACGAGATGTGATCACAGTGGTGACTGGAGTTCAGACGTGTGCTCTTCCGATCT | 100 |
|  | 5'K304 | CAAGCAGAAGACGGCATACGAGATACAGCTGTGACAGTGACTGGAGTTCAGACGTGTGCTCTTCCGATCT | 100 |
|  | 5'K305 | CAAGCAGAAGACGGCATACGAGATCACGCGGGGTCAGTGACTGGAGTTCAGACGTGTGCTCTTCCGATCT | 100 |
|  | 5'K306 | CAAGCAGAAGACGGCATACGAGATGTTCGACCAATTGTGACTGGAGTTCAGACGTGTGCTCTTCCGATCT | 100 |
|  | 5'K307 | CAAGCAGAAGACGGCATACGAGATGATGTGTCACACGTGACTGGAGTTCAGACGTGTGCTCTTCCGATCT | 100 |
|  | 5'K308 | CAAGCAGAAGACGGCATACGAGATAGCACACTGTGTGTGACTGGAGTTCAGACGTGTGCTCTTCCGATCT | 100 |
|  | 5'K309 | CAAGCAGAAGACGGCATACGAGATCCTACAGTTGTCGTGACTGGAGTTCAGACGTGTGCTCTTCCGATCT | 100 |
|  | 5'K310 | CAAGCAGAAGACGGCATACGAGATTGAGGTCAACAGGTGACTGGAGTTCAGACGTGTGCTCTTCCGATCT | 100 |
|  | 5'K311 | CAAGCAGAAGACGGCATACGAGATGTACACGATGTGGTGACTGGAGTTCAGACGTGTGCTCTTCCGATCT | 100 |
|  | 5'K312 | CAAGCAGAAGACGGCATACGAGATACGTGTAGCACAGTGACTGGAGTTCAGACGTGTGCTCTTCCGATCT | 100 |
|  | 5'K313 | CAAGCAGAAGACGGCATACGAGATAGAACATTTGTTGTGACTGGAGTTCAGACGTGTGCTCTTCCGATCT | 100 |
|  | 5'K314 | CAAGCAGAAGACGGCATACGAGATGAGTGGCCAAACGTGACTGGAGTTCAGACGTGTGCTCTTCCGATCT | 100 |
|  | 5'K315 | CAAGCAGAAGACGGCATACGAGATGTGTACAGCATGGTGACTGGAGTTCAGACGTGTGCTCTTCCGATCT | 100 |
|  | 5'K316 | CAAGCAGAAGACGGCATACGAGATACACGTGATGCAGTGACTGGAGTTCAGACGTGTGCTCTTCCGATCT | 100 |
|  | 5'K317 | CAAGCAGAAGACGGCATACGAGATGTGACACTGACAGTGACTGGAGTTCAGACGTGTGCTCTTCCGATCT | 100 |
|  | 5'K318 | CAAGCAGAAGACGGCATACGAGATACAGTGTCAGTGGTGACTGGAGTTCAGACGTGTGCTCTTCCGATCT | 100 |
|  | 5'K319 | CAAGCAGAAGACGGCATACGAGATAGCTGTGTGACAGTGACTGGAGTTCAGACGTGTGCTCTTCCGATCT | 100 |
|  | 5'K320 | CAAGCAGAAGACGGCATACGAGATGATCACACAGTGGTGACTGGAGTTCAGACGTGTGCTCTTCCGATCT | 100 |
|  | 5'K321 | CAAGCAGAAGACGGCATACGAGATGTGTGACATGCAGTGACTGGAGTTCAGACGTGTGCTCTTCCGATCT | 100 |
|  | 5'K322 | CAAGCAGAAGACGGCATACGAGATACACAGTGCATGGTGACTGGAGTTCAGACGTGTGCTCTTCCGATCT | 100 |
|  | 5'K323 | CAAGCAGAAGACGGCATACGAGATGTGTGACTACAGGTGACTGGAGTTCAGACGTGTGCTCTTCCGATCT | 100 |
|  | 5'K324 | CAAGCAGAAGACGGCATACGAGATACACAGTCGTGAGTGACTGGAGTTCAGACGTGTGCTCTTCCGATCT | 100 |
|  | 5'K325 | CAAGCAGAAGACGGCATACGAGATACAGTGTGTCAGGTGACTGGAGTTCAGACGTGTGCTCTTCCGATCT | 100 |
|  | 5'K326 | CAAGCAGAAGACGGCATACGAGATGTGACACACTGAGTGACTGGAGTTCAGACGTGTGCTCTTCCGATCT | 100 |
|  | 5'K327 | CAAGCAGAAGACGGCATACGAGATTTTGTTCACGACGTGACTGGAGTTCAGACGTGTGCTCTTCCGATCT | 100 |
|  | 5'K328 | CAAGCAGAAGACGGCATACGAGATACACACGTGATGGTGACTGGAGTTCAGACGTGTGCTCTTCCGATCT | 100 |
|  | 5'K329 | CAAGCAGAAGACGGCATACGAGATAGTCAGTGTGCAGTGACTGGAGTTCAGACGTGTGCTCTTCCGATCT | 100 |
|  | 5'K330 | CAAGCAGAAGACGGCATACGAGATGACTGACACATGGTGACTGGAGTTCAGACGTGTGCTCTTCCGATCT | 100 |
|  | 5'K331 | CAAGCAGAAGACGGCATACGAGATCGAAGTTGTTCAGTGACTGGAGTTCAGACGTGTGCTCTTCCGATCT | 100 |
|  | 5'K332 | CAAGCAGAAGACGGCATACGAGATTATGACCAACTGGTGACTGGAGTTCAGACGTGTGCTCTTCCGATCT | 100 |
|  | 5'K333 | CAAGCAGAAGACGGCATACGAGATTGAGGGGGACAAGTGACTGGAGTTCAGACGTGTGCTCTTCCGATCT | 100 |
|  | 5'K334 | CAAGCAGAAGACGGCATACGAGATCCTACAAATTGTGTGACTGGAGTTCAGACGTGTGCTCTTCCGATCT | 100 |
|  | 5'K335 | CAAGCAGAAGACGGCATACGAGATTTGTATCACCTGGTGACTGGAGTTCAGACGTGTGCTCTTCCGATCT | 100 |
|  | 5'K336 | CAAGCAGAAGACGGCATACGAGATACAAGCGGTTACGTGACTGGAGTTCAGACGTGTGCTCTTCCGATCT | 100 |
|  | 5'K337 | CAAGCAGAAGACGGCATACGAGATGTGTACACGTAGGTGACTGGAGTTCAGACGTGTGCTCTTCCGATCT | 100 |
|  | 5'K338 | CAAGCAGAAGACGGCATACGAGATACACGTGTACGAGTGACTGGAGTTCAGACGTGTGCTCTTCCGATCT | 100 |
|  | 5'K339 | CAAGCAGAAGACGGCATACGAGATAATCGGTTGTCAGTGACTGGAGTTCAGACGTGTGCTCTTCCGATCT | 100 |
|  | 5'K340 | CAAGCAGAAGACGGCATACGAGATTGATACCCCATTGTGACTGGAGTTCAGACGTGTGCTCTTCCGATCT | 100 |
|  | 5'K341 | CAAGCAGAAGACGGCATACGAGATCGAAATGTTTGAGTGACTGGAGTTCAGACGTGTGCTCTTCCGATCT | 100 |
|  | 5'K342 | CAAGCAGAAGACGGCATACGAGATTATGTCCACACTGTGACTGGAGTTCAGACGTGTGCTCTTCCGATCT | 100 |
|  | 5'K343 | CAAGCAGAAGACGGCATACGAGATGACACTGATGTGGTGACTGGAGTTCAGACGTGTGCTCTTCCGATCT | 100 |
|  | 5'K344 | CAAGCAGAAGACGGCATACGAGATAGTGTCAGCACAGTGACTGGAGTTCAGACGTGTGCTCTTCCGATCT | 100 |
|  | 5'K345 | CAAGCAGAAGACGGCATACGAGATTGTTCACCTATGGTGACTGGAGTTCAGACGTGTGCTCTTCCGATCT | 100 |
|  | 5'K346 | CAAGCAGAAGACGGCATACGAGATACCAGTTGATACGTGACTGGAGTTCAGACGTGTGCTCTTCCGATCT | 100 |
|  | 5'K347 | CAAGCAGAAGACGGCATACGAGATACGTACGTGTACGTGACTGGAGTTCAGACGTGTGCTCTTCCGATCT | 100 |
|  | 5'K348 | CAAGCAGAAGACGGCATACGAGATGTACGTACACGTGTGACTGGAGTTCAGACGTGTGCTCTTCCGATCT | 100 |
|  |  |  |  |
|  |  |  |  |
|  |  |  |  |
| 3' primer (for the I5 index) | | |  |
|  | Name | Sequence | Final concentration (nmol/L) |
|  | 3'H501 | AATGATACGGCGACCACCGAGATCTACACGTACAGTCACGTACACTCTTTCCCTACACGACGCTCTTCCGATCT | 100 |
|  | 3'H502 | AATGATACGGCGACCACCGAGATCTACACACGTGACTGTACACACTCTTTCCCTACACGACGCTCTTCCGATCT | 100 |
|  | 3'H503 | AATGATACGGCGACCACCGAGATCTACACGTACGTGTGACAACACTCTTTCCCTACACGACGCTCTTCCGATCT | 100 |
|  | 3'H504 | AATGATACGGCGACCACCGAGATCTACACACGTACACAGTGACACTCTTTCCCTACACGACGCTCTTCCGATCT | 100 |
|  | 3'H505 | AATGATACGGCGACCACCGAGATCTACACACAGTCAGTGTGACACTCTTTCCCTACACGACGCTCTTCCGATCT | 100 |
|  | 3'H506 | AATGATACGGCGACCACCGAGATCTACACGTGACTGACACAACACTCTTTCCCTACACGACGCTCTTCCGATCT | 100 |
|  | 3'H507 | AATGATACGGCGACCACCGAGATCTACACACGTAGTGTCACACACTCTTTCCCTACACGACGCTCTTCCGATCT | 100 |
|  | 3'H508 | AATGATACGGCGACCACCGAGATCTACACGTACGACACTGTACACTCTTTCCCTACACGACGCTCTTCCGATCT | 100 |
|  | 3'H509 | AATGATACGGCGACCACCGAGATCTACACGACATGTGTCACACACTCTTTCCCTACACGACGCTCTTCCGATCT | 100 |
|  | 3'H510 | AATGATACGGCGACCACCGAGATCTACACAGTGCACACTGTACACTCTTTCCCTACACGACGCTCTTCCGATCT | 100 |
|  | 3'H511 | AATGATACGGCGACCACCGAGATCTACACGTAGCACATGTGACACTCTTTCCCTACACGACGCTCTTCCGATCT | 100 |
|  | 3'H512 | AATGATACGGCGACCACCGAGATCTACACACGATGTGCACAACACTCTTTCCCTACACGACGCTCTTCCGATCT | 100 |
|  | 3'H513 | AATGATACGGCGACCACCGAGATCTACACGTACACGTAGTGACACTCTTTCCCTACACGACGCTCTTCCGATCT | 100 |
|  | 3'H514 | AATGATACGGCGACCACCGAGATCTACACACGTGTACGACAACACTCTTTCCCTACACGACGCTCTTCCGATCT | 100 |
|  | 3'H515 | AATGATACGGCGACCACCGAGATCTACACACGACTGTGTGAACACTCTTTCCCTACACGACGCTCTTCCGATCT | 100 |
|  | 3'H516 | AATGATACGGCGACCACCGAGATCTACACGTAGTCACACAGACACTCTTTCCCTACACGACGCTCTTCCGATCT | 100 |
|  | 3'H517 | AATGATACGGCGACCACCGAGATCTACACGTGTACACGACTACACTCTTTCCCTACACGACGCTCTTCCGATCT | 100 |
|  | 3'H518 | AATGATACGGCGACCACCGAGATCTACACACACGTGTAGTCACACTCTTTCCCTACACGACGCTCTTCCGATCT | 100 |
|  | 3'H519 | AATGATACGGCGACCACCGAGATCTACACAGTGTGTCACAGACACTCTTTCCCTACACGACGCTCTTCCGATCT | 100 |
|  | 3'H520 | AATGATACGGCGACCACCGAGATCTACACGACACACTGTGAACACTCTTTCCCTACACGACGCTCTTCCGATCT | 100 |
|  | 3'H521 | AATGATACGGCGACCACCGAGATCTACACAAATGTTTCACTACACTCTTTCCCTACACGACGCTCTTCCGATCT | 100 |
|  | 3'H522 | AATGATACGGCGACCACCGAGATCTACACGGGCAAACGTTCACACTCTTTCCCTACACGACGCTCTTCCGATCT | 100 |
|  | 3'H523 | AATGATACGGCGACCACCGAGATCTACACTGTGTAACAGGAACACTCTTTCCCTACACGACGCTCTTCCGATCT | 100 |
|  | 3'H524 | AATGATACGGCGACCACCGAGATCTACACCCCAATTTTCCGACACTCTTTCCCTACACGACGCTCTTCCGATCT | 100 |
|  | 3'H525 | AATGATACGGCGACCACCGAGATCTACACGTGTACAGTCACACACTCTTTCCCTACACGACGCTCTTCCGATCT | 100 |
|  | 3'H526 | AATGATACGGCGACCACCGAGATCTACACACACGTGACTGTACACTCTTTCCCTACACGACGCTCTTCCGATCT | 100 |
|  | 3'H527 | AATGATACGGCGACCACCGAGATCTACACGACATGTGCACTACACTCTTTCCCTACACGACGCTCTTCCGATCT | 100 |
|  | 3'H528 | AATGATACGGCGACCACCGAGATCTACACAGTGCACATGTCACACTCTTTCCCTACACGACGCTCTTCCGATCT | 100 |
|  | 3'H529 | AATGATACGGCGACCACCGAGATCTACACGGGGCACATTACACACTCTTTCCCTACACGACGCTCTTCCGATCT | 100 |
|  | 3'H530 | AATGATACGGCGACCACCGAGATCTACACACCCGGGTCAGTACACTCTTTCCCTACACGACGCTCTTCCGATCT | 100 |
|  | 3'H531 | AATGATACGGCGACCACCGAGATCTACACACAGTGTCACGTACACTCTTTCCCTACACGACGCTCTTCCGATCT | 100 |
|  | 3'H532 | AATGATACGGCGACCACCGAGATCTACACGTGACACTGTACACACTCTTTCCCTACACGACGCTCTTCCGATCT | 100 |
|  | 3'H533 | AATGATACGGCGACCACCGAGATCTACACATGTGTGAATCAACACTCTTTCCCTACACGACGCTCTTCCGATCT | 100 |
|  | 3'H534 | AATGATACGGCGACCACCGAGATCTACACGACCAAAGGCGTACACTCTTTCCCTACACGACGCTCTTCCGATCT | 100 |
|  | 3'H535 | AATGATACGGCGACCACCGAGATCTACACACGTGTACACGTACACTCTTTCCCTACACGACGCTCTTCCGATCT | 100 |
|  | 3'H536 | AATGATACGGCGACCACCGAGATCTACACGTACACGTGTACACACTCTTTCCCTACACGACGCTCTTCCGATCT | 100 |
|  | 3'H537 | AATGATACGGCGACCACCGAGATCTACACGTACAGGTTAAAACACTCTTTCCCTACACGACGCTCTTCCGATCT | 100 |
|  | 3'H538 | AATGATACGGCGACCACCGAGATCTACACAAGTTCCAATGGACACTCTTTCCCTACACGACGCTCTTCCGATCT | 100 |
|  | 3'H539 | AATGATACGGCGACCACCGAGATCTACACTGTACCACGCGGACACTCTTTCCCTACACGACGCTCTTCCGATCT | 100 |
|  | 3'H540 | AATGATACGGCGACCACCGAGATCTACACCCAGTTTGCGCAACACTCTTTCCCTACACGACGCTCTTCCGATCT | 100 |
|  | 3'H541 | AATGATACGGCGACCACCGAGATCTACACCGGACGGTTAAAACACTCTTTCCCTACACGACGCTCTTCCGATCT | 100 |
|  | 3'H542 | AATGATACGGCGACCACCGAGATCTACACGACGTAAACTTTACACTCTTTCCCTACACGACGCTCTTCCGATCT | 100 |
|  | 3'H543 | AATGATACGGCGACCACCGAGATCTACACATTAAATTTGAAACACTCTTTCCCTACACGACGCTCTTCCGATCT | 100 |
|  | 3'H544 | AATGATACGGCGACCACCGAGATCTACACGCATGTCAAAGGACACTCTTTCCCTACACGACGCTCTTCCGATCT | 100 |
|  | 3'H545 | AATGATACGGCGACCACCGAGATCTACACGTACACAGTCGTACACTCTTTCCCTACACGACGCTCTTCCGATCT | 100 |
|  | 3'H546 | AATGATACGGCGACCACCGAGATCTACACACGTGTGACTACACACTCTTTCCCTACACGACGCTCTTCCGATCT | 100 |
|  | 3'H547 | AATGATACGGCGACCACCGAGATCTACACGAGGTGTGCAAAACACTCTTTCCCTACACGACGCTCTTCCGATCT | 100 |
|  | 3'H548 | AATGATACGGCGACCACCGAGATCTACACATCAAACAGGTTACACTCTTTCCCTACACGACGCTCTTCCGATCT | 100 |
