# Supplementary material for: A highly specific and sensitive massive parallel sequencer-based test for somatic mutations in non-small cell lung cancer
Source: PLoS One. 2017 Apr 27;12(4):e0176525. doi: 10.1371/journal.pone.0176525 (PMC5407820; doi:10.1371/journal.pone.0176525)
Supplement: S5 Table — The results were sorted first by the type of mutation and then the calculated cancer-cell content. (DOCX) [file pone.0176525.s008.docx]

| No. |  | Mutation | Sample source | Calculated cancer-cell content (%) |
| --- | --- | --- | --- | --- |
| 1 |  | *EGFR* E746-A750del Type1 ^a^ | Pleural effusion | 100.0 |
| 2 |  | *EGFR* E746-A750del Type1 | Lung Cancer Tissue | 100.0 |
| 3 |  | *EGFR* E746-A750del Type1 | Not described | 100.0 |
| 4 |  | *EGFR* E746-A750del Type1 | Bronchial brushing | 99.6 |
| 5 |  | *EGFR* E746-A750del Type1 | Lung Cancer Tissue | 48.0 |
| 6 |  | *EGFR* E746-A750del Type1 | Bronchial brushing | 10.5 |
| 7 |  | *EGFR* E746-A750del Type1 | Bronchial brushing | 8.6 |
| 8 |  | *EGFR* E746-A750del Type1 | Bronchial brushing | 6.1 |
| 9 |  | *EGFR* E746-A750del Type2^b^ | Lung Cancer Tissue | 34.2 |
| 10 |  | *EGFR* E746-A750del Type2  + *EGFR* T790M | Pleural effusion | 5.7 + 1.5 |
| 11 |  | *EGFR* E746-A750del Type1  + *EGFR* T790M | Pleural effusion | 78.6 + 20.9 |
| 12 |  | *EGFR* L858R | Lung Cancer Tissue | 100.0 |
| 13 |  | *EGFR* L858R | Bronchial brushing | 100.0 |
| 14 |  | *EGFR* L858R | Lung Cancer Tissue | 73.6 |
| 15 |  | *EGFR* L858R | Bronchial brushing | 60.0 |
| 16 |  | *EGFR* L858R | Lung Cancer Tissue | 45.1 |
| 17 |  | *EGFR* L858R | Pleural effusion | 36.0 |
| 18 |  | *EGFR* L858R | Not described | 35.4 |
| 19 |  | *EGFR* L858R | Lung Cancer Tissue | 34.9 |
| 20 |  | *EGFR* L858R | Lung Cancer Tissue | 33.7 |
| 21 |  | *EGFR* L858R | Bronchial brushing | 25.1 |
| 22 |  | *EGFR* L858R | Not described | 8.1 |
| 23 |  | *EGFR* L858R | Bronchial brushing | 7.7 |
| 24 |  | *EGFR* L858R | Bronchial brushing | 7.2 |
| 25 |  | *EGFR* L858R | Pleural effusion | 3.3 |
| 26 |  | *EGFR* L858R | Bronchial brushing | 1.2 |
| 27 |  | *EGFR* L858R^c^ | Lung Cancer Tissue | 0.8 |
| 28 |  | *KRAS* G12V | Not described | 100.0 |
| 29 |  | *KRAS* G12V | Bronchial brushing | 93.9 |
| 30 |  | *KRAS* G12V | Lung Cancer Tissue | 24.3 |
| 31 |  | *KRAS* G12V | Bronchial brushing | 7.5 |
| 32 |  | *KRAS* G12V | Lung Cancer Tissue | 3.6 |
| 33 |  | *KRAS* G12V | Bronchial brushing | 1.4 |
| 34 |  | *KRAS* G12S | Bronchial brushing | 10.0 |
| 35 |  | *KRAS* G12R | Not described | 52.1 |
| 36 |  | *KRAS* G12D | Lung Cancer Tissue | 78.0 |
| 37 |  | *KRAS* G12D | Bronchial brushing | 59.3 |
| 38 |  | *KRAS* G12D | Bronchial brushing | 7.0 |
| 39 |  | *KRAS* G12D | Bronchial brushing | 1.8 |
| 40 |  | *KRAS* G12A | Lung Cancer Tissue | 81.0 |
| 41 |  | *KRAS* G12A | Bronchial brushing | 12.2 |
| 42 |  | *KRAS* G12A  + *KRAS* G12C | Lung Cancer Tissue | 46.7 + 2.1 |
| 43 |  | *KRAS* G12C | Lung Cancer Tissue | 16.8 |
| 44 |  | *KRAS* G13D | Bronchial brushing | 100.0 |
| 45 |  | *KRAS* Q61H 1 | Pleural effusion | 2.1 |
| 46 |  | *BRAF* G469A | Lung Cancer Tissue | 32.5 |
| 47 |  | *BRAF* G466V | Pleural effusion | 1.4 |
| 48 |  | Negative | Bronchial brushing |  |
| 49 |  | Negative | Bronchial brushing |  |
| 50 |  | Negative | Not described |  |
| 51 |  | Negative | Not described |  |
| 52 |  | Negative | Lung Cancer Tissue |  |
| 53 |  | Negative | Bronchial brushing |  |
| 54 |  | Negative | Lung Cancer Tissue |  |
| 55 |  | Negative | Lung Cancer Tissue |  |
| 56 |  | Negative | Bronchial brushing |  |
| 57 |  | Negative | Bronchial brushing |  |
| 58 |  | Negative | Lung Cancer Tissue |  |
| 59 |  | Negative | Not described |  |
| 60 |  | Negative | Lung Cancer Tissue |  |
| 61 |  | Negative | Lung Cancer Tissue |  |
| 62 |  | Negative | Lung Cancer Tissue |  |
| 63 |  | Negative | Lung Cancer Tissue |  |
| 64 |  | Negative | Lung Cancer Tissue |  |
| 65 |  | Negative | Lung Cancer Tissue |  |
| 66 |  | Negative | Lung Cancer Tissue |  |
| 67 |  | Negative | Pleural effusion |  |
| 68 |  | Negative | Bronchial brushing |  |
| 69 |  | Negative | Bronchial brushing |  |
| 70 |  | Negative | Lung Cancer Tissue |  |
| 71 |  | Negative | Bronchial brushing |  |
| 72 |  | Negative | Lung Cancer Tissue |  |
| 73 |  | Negative | Not described |  |
| 74 |  | Negative | Lung Cancer Tissue |  |
| 75 |  | Negative | Lung Cancer Tissue |  |
| 76 |  | Negative | Lung Cancer Tissue |  |
| 77 |  | Negative | Lung Cancer Tissue |  |
| 78 |  | Negative | Lung Cancer Tissue |  |
| 79 |  | Negative | Not described |  |
| 80 |  | Negative | Not described |  |
| 81 |  | Negative | Lung Cancer Tissue |  |
| 82 |  | Negative | Lung Cancer Tissue |  |
| 83 |  | Negative | Pleural effusion |  |
| 84 |  | Negative | Bronchial brushing |  |
| 85 |  | Negative | Bronchial brushing |  |
| 86 |  | Negative | Bronchial brushing |  |
| 87 |  | Negative | Bronchial brushing |  |
| 88 |  | Negative | Bronchial brushing |  |
| 89 |  | Negative | Bronchial brushing |  |
| 90 |  | Negative | Bronchial brushing |  |
| 91 |  | Negative | Lung Cancer Tissue |  |
| 92 |  | Negative | Not described |  |
| 93 |  | Negative | Bronchial brushing |  |
| 94 |  | Negative | Bronchial brushing |  |
| 95 |  | Negative | Bronchial brushing |  |
| 96 |  | Not determined | Lung Cancer Tissue |  |

^a^ *EGFR* E746-A750del Type1 indicates *EGFR* 2235_2249delGGAATTAAGAGAAGC. ^b^ *EGFR* E746-A750del Type2 indicates *EGFR* 2236_2250delGAATTAAGAGAAGCA.

^c^ Negative for mutation when tested by the PNA-LNA PCR clamp method.
