# Supplementary material for: A highly specific and sensitive massive parallel sequencer-based test for somatic mutations in non-small cell lung cancer
Source: PLoS One. 2017 Apr 27;12(4):e0176525. doi: 10.1371/journal.pone.0176525 (PMC5407820; doi:10.1371/journal.pone.0176525)
Supplement: S6 Table — The results are presented by the type of mutations, and sorted by either the percentage of the cells harboring the mutation in the samples or the number of reads for the OAZ1 gene (DOC). (DOCX) [file pone.0176525.s009.docx]

| Mutations detected by the DNA part of the MINtS | | |  |  |  |
| --- | --- | --- | --- | --- | --- |
| Sample number | Mutations | Mutation type | Cells harboring mutations (%) | *OAZ1* gene reads (n) |  |
| 1 | *EGFR* exon 19 deletion | Exon 19 del (E747_A750del type 2) | 100.0 | 144003 |  |
| 2 |  | Exon 19 del (E746_A750del type 2) | 98.4 | 21626 |  |
| 3 |  | Exon 19 del (E746-A750del type2) | 98.2 | 65203 |  |
| 4 |  | Exon 19 del (L747_S752del_P753S) | 69.7 | 21858 |  |
| 5 |  | Exon 19 del (E746-A750del type2) | 67.3 | 29419 |  |
| 6 |  | Exon 19 del (L747-S752del P753S) | 63.4 | 120382 |  |
| 7 |  | Exon 19 del (L747-T751dell) | 45.3 | 167055 |  |
| 8 |  | Exon 19 delL(747-S752del P753S) | 39.9 | 8149 |  |
| 9 |  | Exon 19 del (E746_A750del type 1) | 37.0 | 53325 |  |
| 10 |  | Exon 19 del (L747-S749del A750P) | 22.2 | 143969 |  |
| 11 | *EGFR* L858R mutation | L858R | 100.0 | 72420 |  |
| 12 |  | L858R | 85.7 | 154403 |  |
| 13 |  | L858R | 74.0 | 85506 |  |
| 14 |  | L858R | 72.0 | 19653 |  |
| 15 |  | L858R | 71.9 | 205399 |  |
| 16 |  | L858R | 69.8 | 186074 |  |
| 17 |  | L858R | 61.4 | 51550 |  |
| 18 |  | L858R | 55.2 | 154529 |  |
| 19 |  | L858R | 54.9 | 168076 |  |
| 20 |  | L858R | 51.3 | 103170 |  |
| 21 |  | L858R | 47.9 | 73119 |  |
| 22 |  | L858R | 45.7 | 20822 |  |
| 23 |  | L858R | 44.2 | 140610 |  |
| 24 |  | L858R | 41.1 | 131274 |  |
| 25 |  | L858R | 39.9 | 20885 |  |
| 26 |  | L858R | 39.5 | 79910 |  |
| 27 |  | L858R | 36.1 | 65962 |  |
| 28 |  | L858R | 34.7 | 25771 |  |
| 29 |  | L858R | 33.7 | 29450 |  |
| 30 |  | L858R | 31.7 | 29724 |  |
| 31 |  | L858R | 28.7 | 60613 |  |
| 32 |  | L858R | 27.1 | 22528 |  |
| 33 |  | L858R | 25.7 | 195608 |  |
| 34 |  | L858R | 22.0 | 160104 |  |
| 35 |  | L858R | 20.6 | 21217 |  |
| 36 |  | L858R | 20.0 | 7892 |  |
| 37 |  | L858R | 19.3 | 21881 |  |
| 38 |  | L858R | 16.8 | 23125 |  |
| 39 |  | L858R | 5.9 | 65162 |  |
|  |  |  |  |  |  |
| 40 | *EGFR* exon 18 minor mutations | G719A | 100.0 | 194556 |  |
| 41 |  | G719C | 100.0 | 20938 |  |
| 42 |  | G719S | 48.9 | 17010 |  |
| 43 |  | G719A | 37.6 | 78495 |  |
|  |  | G719S | 37.2 | 20330 |  |
|  |  |  |  |  |  |
| 44 | *EGFR* exon 21 minor mutations | L861Q | 58.9 | 106373 |  |
| 45 |  | L861Q | 47.8 | 18402 |  |
| 46 |  | L861Q | 17.4 | 21772 |  |
|  |  |  |  |  |  |
| 47 | With *EGFR* T790M second mutation | L858R + T790M | 31.0 + 37.0 | 143746 |  |
| 48 |  | L858R + T790M | 84.5 + 88.0 | 81859 |  |
|  |  |  |  |  |  |
|  |  |  |  |  |  |
| 49 | *KRAS* mutations in codons 12 and 13 | G12A | 100.0 | 62817 |  |
| 50 |  | G12C | 88.2 | 118695 |  |
| 51 |  | G12C | 62.9 | 167807 |  |
| 52 |  | G12C | 47.1 | 22710 |  |
| 53 |  | G12C | 47.1 | 33703 |  |
| 54 |  | G12C | 43.2 | 78305 |  |
| 55 |  | G12C | 41.0 | 15561 |  |
| 56 |  | G12D | 63.9 | 164004 |  |
| 57 |  | G12V | 62.4 | 22158 |  |
| 58 |  | G12D | 50.1 | 27163 |  |
| 59 |  | G12V | 40.7 | 161077 |  |
| 60 |  | G12D | 32.5 | 16460 |  |
| 61 |  | G12V | 32.0 | 68491 |  |
| 62 |  | G12C | 23.8 | 19132 |  |
| 63 |  | G12V | 21.8 | 20747 |  |
| 64 |  | G12D | 19.2 | 15971 |  |
| 65 |  | G12V | 18.6 | 88048 |  |
| 66 |  | G12V | 16.1 | 120382 |  |
| 67 |  | G12D | 6.4 | 170278 |  |
| 68 |  | G13D | 6.4 | 84902 |  |
| 69 |  | G12D | 5.8 | 51805 |  |
| 70 |  | G12A | 1.1 | 189922 |  |
| 71 | *KRAS* mutations in codon 61 | Q61H | 7.4 | 24312 |  |
| 72 |  | Q61K | 1.6 | 4742 |  |
|  |  |  |  |  |  |
| 73 | *BRAF* mutations | G469A | 58.6 | 86593 |  |
| 74 |  | V600E | 19.7 | 33897 |  |
|  |  |  |  |  |  |
| 75 | *ERBB2* mutation | YVMA776-779ins | 30.8 | 27212 |  |
| 76 |  | YVMA776-779ins | 53.5 | 117866 |  |
| 77 |  | YVMA776-779ins | 31.9 | 12340 |  |
|  |  |  |  |  |  |
|  |  |  |  |  |  |
| Mutations detected by the RNA part of the MINtS | | |  |  |  |
|  | RNA fusion genes |  | Fusion gene reads (n) | *OAZ1* gene reads (n) | Read ratio (Fusion/*OAZ1*) |
| 78 | *ALK* fusion gene | *EML4*-*ALK* (variant 1) | 120241 | 119756 | 1 |
| 79 |  | *EML4*-*ALK* (variant 1) | 69603 | 166967 | 0.42 |
| 80 |  | *EML4*-*ALK* (variant 1) | 28677 | 30187 | 0.95 |
| 81 | *ROS1* fusion gene | *CD74*-*ROS1* (C6-R34) | 21754 | 23579 | 0.92 |
| 82 |  | *EZR*-*ROS1* (E10-R34) | 10327 | 26061 | 0.4 |
| 83 | *RET* fusion gene | *KIF5B*-*RET* (K15-R12) | 178043 | 136494 | 1.3 |
| 84 |  | *KIF5B*-*RET* (K15_R12) | 34165 | 62902 | 0.54 |
|  |  |  |  |  |  |
|  | Mutation not detected |  |  | *OAZ1* gene reads (n) |  |
| 85 |  | - |  | 225066 |  |
| 86 |  | - |  | 217234 |  |
| 87 |  | - |  | 177750 |  |
| 88 |  | - |  | 175940 |  |
| 89 |  | - |  | 174759 |  |
| 90 |  | - |  | 173287 |  |
| 91 |  | - |  | 170990 |  |
| 92 |  | - |  | 163806 |  |
| 93 |  | - |  | 161970 |  |
| 94 |  | - |  | 147102 |  |
| 95 |  | - |  | 146192 |  |
| 96 |  | - |  | 144641 |  |
| 97 |  | - |  | 142676 |  |
| 98 |  | - |  | 137346 |  |
| 99 |  | - |  | 137288 |  |
| 100 |  | - |  | 132334 |  |
| 101 |  | - |  | 120832 |  |
| 102 |  | - |  | 114308 |  |
| 103 |  | - |  | 106381 |  |
| 104 |  | - |  | 94989 |  |
| 105 |  | - |  | 94951 |  |
| 106 |  | - |  | 89642 |  |
| 107 |  | - |  | 88892 |  |
| 108 |  | - |  | 84975 |  |
| 109 |  | - |  | 81300 |  |
| 110 |  | - |  | 79858 |  |
| 111 |  | - |  | 79733 |  |
| 112 |  | - |  | 79135 |  |
| 113 |  | - |  | 75984 |  |
| 114 |  | - |  | 75883 |  |
| 115 |  | - |  | 75826 |  |
| 116 |  | - |  | 75148 |  |
| 117 |  | - |  | 73685 |  |
| 118 |  | - |  | 73605 |  |
| 119 |  | - |  | 72092 |  |
| 120 |  | - |  | 72087 |  |
| 121 |  | - |  | 69772 |  |
| 122 |  | - |  | 69182 |  |
| 123 |  | - |  | 67767 |  |
| 124 |  | - |  | 66485 |  |
| 125 |  | - |  | 65541 |  |
| 126 |  | - |  | 65456 |  |
| 127 |  | - |  | 64324 |  |
| 128 |  | - |  | 63376 |  |
| 129 |  | - |  | 54192 |  |
| 130 |  | - |  | 52110 |  |
| 131 |  | - |  | 50747 |  |
| 132 |  | - |  | 50598 |  |
| 133 |  | - |  | 46270 |  |
| 134 |  | - |  | 36520 |  |
| 135 |  | - |  | 32012 |  |
| 136 |  | - |  | 31256 |  |
| 137 |  | - |  | 29617 |  |
| 138 |  | - |  | 29489 |  |
| 139 |  | - |  | 28441 |  |
| 140 |  | - |  | 27575 |  |
| 141 |  | - |  | 26992 |  |
| 142 |  | - |  | 26991 |  |
| 143 |  | - |  | 26745 |  |
| 144 |  | - |  | 26416 |  |
| 145 |  | - |  | 26294 |  |
| 146 |  | - |  | 26088 |  |
| 147 |  | - |  | 25190 |  |
| 148 |  | - |  | 25127 |  |
| 149 |  | - |  | 24912 |  |
| 150 |  | - |  | 24828 |  |
| 151 |  | - |  | 24648 |  |
| 152 |  | - |  | 24512 |  |
| 153 |  | - |  | 24337 |  |
| 154 |  | - |  | 24038 |  |
| 155 |  | - |  | 23895 |  |
| 156 |  | - |  | 23806 |  |
| 157 |  | - |  | 23740 |  |
| 158 |  | - |  | 23621 |  |
| 159 |  | - |  | 23040 |  |
| 160 |  | - |  | 22951 |  |
| 161 |  | - |  | 22524 |  |
| 162 |  | - |  | 22349 |  |
| 163 |  | - |  | 22244 |  |
| 164 |  | - |  | 21670 |  |
| 165 |  | - |  | 21187 |  |
| 166 |  | - |  | 20902 |  |
| 167 |  | - |  | 20768 |  |
| 168 |  | - |  | 20702 |  |
| 169 |  | - |  | 19864 |  |
| 170 |  | - |  | 18887 |  |
| 171 |  | - |  | 18844 |  |
| 172 |  | - |  | 18613 |  |
| 173 |  | - |  | 18287 |  |
| 174 |  | - |  | 17769 |  |
| 175 |  | - |  | 17580 |  |
| 176 |  | - |  | 17219 |  |
| 177 |  | - |  | 17217 |  |
| 178 |  | - |  | 15894 |  |
| 179 |  | - |  | 15867 |  |
| 180 |  | - |  | 15570 |  |
| 181 |  | - |  | 15427 |  |
| 182 |  | - |  | 15055 |  |
| 183 |  | - |  | 15052 |  |
| 184 |  | - |  | 15022 |  |
| 185 |  | - |  | 13664 |  |
| 186 |  | - |  | 10422 |  |
| 187 |  | - |  | 10296 |  |
| 188 |  | - |  | 7149 |  |
|  |  |  |  |  |  |
| 189 | DNA or RNA was degraded | - |  |  |  |
| 190 |  | - |  |  |  |
